# Utilizing remote sensing and field data for geological mapping and polyphase deformation analysis of Um Laseifa ophiolites, Eastern Desert, Egypt

**DOI:** 10.1038/s41598-025-88989-z

**Published:** 2025-02-24

**Authors:** Mahmoud K. Alawy, Mohamed Abdelwahed, Abdel-Kader M. Moghazi, Fathy H. Mohamed, Hossam Khamis, Ali Shebl

**Affiliations:** 1 Department of Geology, Faculty of Science, Alexandria University, Alexandria, 21511 Egypt; 2 Department of Geology, Faculty of Science, Cairo University, Giza, 12613 Egypt; 3 Nuclear Materials Authority, 530, El-Maadi, Cairo, Egypt; 4 Department of Mineralogy and Geology, Faculty of Science and Technology, University of Debrecen, Egyetem ter 1, Debrecen, 4032 Hungary; 5 Department of Geology, Faculty of Science, Tanta University, Tanta, 31527 Egypt

**Keywords:** Remote sensing, Ophiolites, Geological mapping, Deformation phases, Superposed folds, Eastern Desert, Precambrian geology, Structural geology

## Abstract

The Wadi Um Laseifa area, located in the Central Eastern Desert of Egypt, encompasses a range of Neoproterozoic rock units, including ophiolitic mélange, island arc assemblage, and granitic intrusions as well as Miocene clastic deposits. The current research attempts to analyze the structural and lithological characteristics of this area by integrating data from multisource remote sensing (Sentinel 2, Planetscope and hyperspectral PRISMA), along with field and structural relationships, geometrical analysis of structural readings, and petrographic studies. Applying various techniques of remote sensing, such as false color composite (FCC), principal component analysis (PCA), and Minimum noise fraction (MNF), enabled the identification of the structural features over various scales besides accurate lithological discrimination. Data analyses have discriminated the intricate Neoproterozoic rocks into ophiolitic mélange that includes serpentinites, meta-pyroxenites, metagabbro, chert and mélange matrix, island arc assemblage comprising metavolcanics, metavolcano-sedimentary rocks and hornblende schist, and monzogranite and granodiorite intrusions. These rocks have been affected by a thrust stack of three major faults striking NW-SE to NNW-SSE and dipping steeply to the SW. There are two prominent folds represented by a major anticline affecting the island arc metavolcano-sedimentary rocks and a major syncline affecting the ophiolitic rocks. Both folds possess axial planes striking NW-SE and gently plunging NW fold axes. The area is also intersected by E-W or ENE-WSW strike-slip faults, along with major NW-SE normal faults that controlled the distribution of the Miocene clastic deposits. Geometrical analysis has identified three ductile deformation phases: D_1_ is marked by NW-SE isoclinal folds; D_2_ produced NW-SE major folds and thrust faults that are coaxial with D_1_; and D_3_ led to the formation of NE-SW open folds. The multisource remote sensing analysis that has been carried out in this work illustrated the efficacy of the employed methodology in conducting thorough geological analyses and strongly advocates for its application in analogous studies in arid environments.

## Introduction

Various environmental and geological applications, such as mineral exploration^1–4^, finding groundwater reserves^5^, and unstable ground analysis^6^, depend mainly on accurate geological mapping^7^. An integral part of geological mapping involves a systematic investigation and assessment of geological features, structures, and rock units^8–10^. The fundamental objectives of geological mapping are lithological discrimination, tracing minerals, and revealing other geological features that are present in a certain area^11^. Detailed geological mapping also yields insights into geologic structures such as folds and faults. Multiscale mapping and tracing of geologic structures particularly in highly deformed areas can provide, with alternative tools, valuable insights for understanding deformation history and interpreting the local tectonic setting of a certain area. Traditionally, geological mapping has primarily been conducted on land, using data gathered from outcrop-scale observations. However, the use of remote sensing techniques for mapping has seen a notable increase in popularity in recent years^11–16^. Geologically, remote sensing data have demonstrated significant utilities in a range of applications including lithologic mapping^13–16,29,30^, mineral exploration^31,32^, geomorphological mapping^33–36^, and structural interpretations^37^. Consequently, the present study employed a variety of datasets encompassing different spatial resolutions, ranging from 3 m to 30 m pixel size, as well as varying spectral characteristics, from multispectral to hyperspectral. This approach facilitates a comprehensive depiction of the lithological and structural features in this study.

**Fig. 1:**
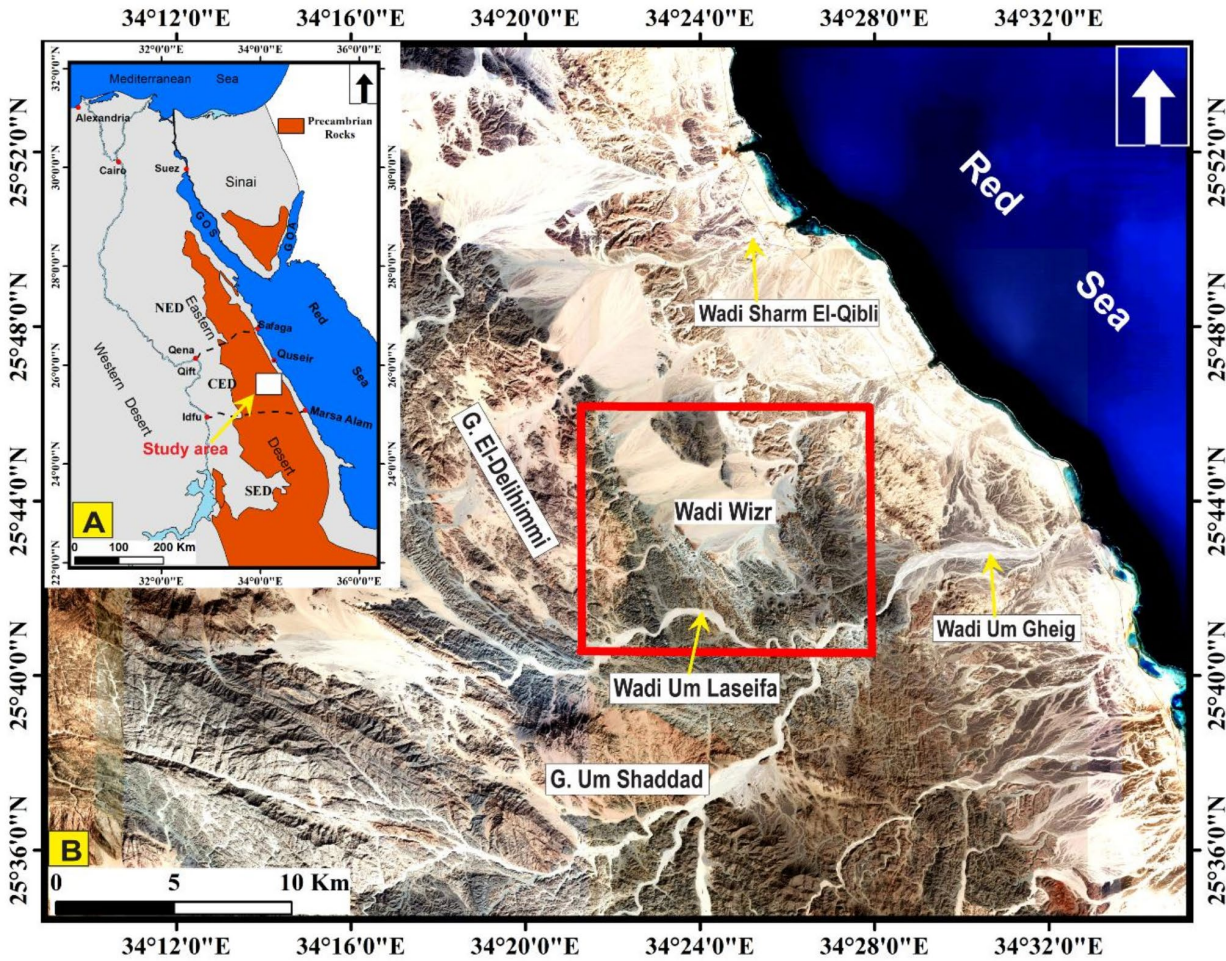
(**A**) Location of the study area along the Red Sea Coast, (NED, Northern Eastern Desert; CED, Central Eastern Desert; SED, Southern Eastern Desert), (**B**) Natural colour high resolution satellite image, extracted from Google Earth, showing the study area (Red box) and the surrounding areas. The figure was created by ArcGIS Desktop 10.8. https://www.esri.com/en-us/arcgis/products/arcgis-desktop/overview, and SmartSketch v. 4.0 software; https://smartsketch.software.informer.com/4.0/.

The study area known as Um Laseifa, situated in the Central Eastern Desert (CED) of Egypt (Fig. 1), comprises Neoproterozoic basement rocks of the Arabian Nubian Shield (ANS). These rocks were formed during the Pan-African orogenic cycle between 850 and 550 Ma^38–43^. The ANS represents the northern segment of a great collision zone referred to as the “East African Orogen” (EAO)^40^, which developed in the Neoproterozoic when East and West Gondwana collided to form the supercontinent Gondwana^40,44,45^. The EAO is subdivided into the ANS in the north that is composed largely of juvenile Neoproterozoic crust^40,46,47^ and the Mozambique Belt to the south^48–51^. The crustal and tectonic evolution of the ANS is very complex and took place through the following stages^40,52^: (1) break-up of Rodina and opening of the Mozambique Ocean (870 − 800 Ma), (2) sea floor spreading and arc-back-arc formation followed by terrane accretion (800 − 670 Ma), (3) continental collision forming the EAO (650 − 600 Ma) and beginning of magmatism and volcano-sedimentary deposition and (4) continuing shorting, deposition, escape tectonics and orogenic collapse (600 − 550 Ma)^52^. Three major tectonic phases have been recognized within the rocks of the ANS; the oldest was an oceanic phase that encompass remnants of ophiolites and island arcs. The second phase included arc accretion and lithospheric thickening, while the third one was widespread NW-SE extension^53^. These tectonic events and complex lithofacies illustrate the intricate and challenging nature of interpreting the structural framework and identifying lithological units within the study area. Several previous studies, focused primarily on the geological mapping of the study area and its surroundings^54–56^, have confirmed this complexity. Despite these efforts, there remains considerable controversy, particularly regarding the types and locations of major structures, as well as the detailed mapping and nomenclature of the rock units. Furthermore, the deformation phases affecting the rock units have been interpreted differently by various researchers^56–58^. It is observed that most remote sensing efforts in the Eastern Desert have focused primarily on lithological discrimination of Neoproterozoic rocks, with limited application of this technology for tracing major and minor structures, apart from a few recent studies^37^. Tracing structures is crucial, as they closely reflect and relate to outcrop-scale features, providing valuable insights for constructing the structural framework and understanding the deformation history of the study area. Additionally, delineating deformation events is important for gold mineralization, as it is widely accepted that gold deposits are structurally controlled and that ore concentration is influenced by successive deformation phases affecting the gold-bearing rocks^59^.

This work aims to address these challenges by clarifying the structural and lithological contexts of the study area. It represents the first study to integrate Sentinel-2, PlanetScope, and hyperspectral PRISMA data with detailed field observations, structural measurements, geometrical analysis, and petrographic investigations across the area. The objective is to produce an accurate geological map based on this multiscale methodology and diverse datasets. Additionally, the study seeks to elucidate the local deformation history, the structural setting of the exposed rocks, and their relationship to the broader tectonic framework of the surrounding region.

## Geological background and previous work

The prolonged Neoproterozoic crustal evolution of the ANS was marked by the accumulation of Cryogenian (800–650 Ma) island arc metavolcano-sedimentary successions, dismembered ophiolitic nappes and ophiolitic mélange, all intruded by metagabbro-diorite, syn-tectonic older granitoids^40,60^. This was succeeded by an Ediacaran post-collisional stage (630–550 Ma) characterized by the emplacement of calc-alkaline to alkaline magmatism across the shield^60–62^. The Eastern Desert (ED) of Egypt represents the northwestern extension of the ANS, and it was divided into three terranes, namely: Northern Eastern Desert (NED), Central Eastern Desert (CED), and Southern Eastern Desert (SED). The main rock types of the CED are predominantly greenschist-facies ophiolitic rocks and island arc metavolcano-sedimentary sequences^63^. This assemblage represents the oldest recognized units of the CED, with U-Pb zircon ages of ~ 750 Ma^64^. It is interspersed with Cryogenian I-type granitoids and Ediacaran I- and A-type granites and is further disrupted by several Ediacaran magmatic-metamorphic core complexes^65–67^.

Ophiolites and ophiolitic mélanges occupy small surface areas in the CED and SED. These rocks were considered as part of oceanic supracrustal rocks emplaced onto deeper parts of infra-crustal rocks during the time of terrane accretion in the ED^60,68–71^. They were delineated in many areas^72^, such as Fawakhir area^73,74^, Wadi Beririq^75^, Bir Al-Edeid area^76^ and Wadi Semna^77^, Um Salatit-Barramiya area^78^ and Wadi Ghadir^29,79–81^. In areas such as Fawkhir, Ghadir, and Gerf, a complete ophiolite sequences consist of a mantle section, composed of serpentinized ultramafic rocks, and a crustal sequence including layered and isotropic gabbros, sheeted dykes, and either massive or pillow basalts have been documented^74,81–83^. In most other areas, they are dismembered, often missing one or more of their characteristic lithologies^84^ and are found either as a nappe (an intact thrust sheet) or as a mélange (a tectonic mixture of fragments).

The complex tectonic evolution and long deformation history affect the ANS and the ED results in presence of different tectonic regimes (compressional, wrench, and extensional)^85^. These different tectonic regimes created and resulted in presence of different styles of geologic structures. For instance, the SED is characterized by prevailing of thrusts imbricated thrust stacks. The CED is distinguished by a wide variety of geologic structures such as thrusting and thrust-related folds, foliations, lineations, doming, shearing and shear zone-related structures. The NED is marked by a wide variety of extensional faults and shear fractures^86^.

Um Laseifa area lies in the CED of Egypt, at about 50 km to the south of Quseir city (Fig. 1). It is bounded by latitudes 25° 40’ 32.10” and 25° 46’ 7.8” N and longitudes 34° 21’ 21.32” and 34° 27’ 56.8"E. The study area is characterized by Neoproterozoic basement rocks, which include an ophiolitic mélange association, and arc assemblage rocks. The area is also intruded by granitoid rocks, and dykes of variable composition. In addition, dispersing blocks of Phanerozoic rocks represented by Miocene clastic deposits^87^ occur in the northeastern sector.

The study area was mapped differently in previous literatures. Asran^54^ mapped the rock units as only ophiolitic rocks (serpentinites, metagabbros and metabasalts). Hamimi^55^ mapped it as dismembered blocks of metaultramafites set in a matrix of low-grade volcanogenic metasediments and locally highly sheared serpentinites. El Bahariya et al.^58^ and Ibrahim and Cosgrove^88^ mapped the study area as metavolcanics with few exposures of ophiolitic mélange. Hebiry^56^, Abdeen^89^, and Abdeen et al.^90^ described the rock cover as Pan-African nappe complex represented by ophiolitic mélange and related metavolcanic and metasedimentary rocks. Farahat^91^ and Farahat et al.^92^ differentiated that ophiolite nappe to serpentinites, metagabbros and metabasalts). Fowler et al.^57^ differentiated the ophiolitic blocks to ultramafites, gabbros, dolerites, pillow basalts and mélange, where Abdel-Karim and Ahmed^82^ described these blocks as serpentinites, talc-carbonate rocks, meta-pyroxenites, metagabbros, and metabasalts. Beside previous lithologic units, other rock units such as metagabbro-diorite rocks and foliated metavolcanics were identified in the ophiolitic and arc assemblage^93^.

Structurally, Asran^54^ stated that the rocks units are affected by a synclinal fold and several faults of NW trends, whereas El Bahariya et al.^58^ mapped two folds: a syncline and an anticline trending NW and WNW, respectively. In addition, the authors mapped normal faults controlling the contacts between the major rock units. Hebiry^56^ showed that the entire area was affected by four antiformal and synformal structures. Also, the geologic map prepared by Abdeen^89^ and Abdeen et al.^90^ shows some major folds with two orientations; the first is NW-SE to WNW-ESE (predominant) and the other is NE-SW (weakly preserved). The first trend forms a series of NE-verging synformal and antiformal folds whose axial planes are parallel to sub-parallel to SW-dipping oblique-slip faults. Previous efforts also attempted to address the successive deformation events affected on Um Laseifa area. For instance, El Bahariya et al.^58^ identified three phases of deformation and metamorphism. The ductile deformations (D_1_ and D_2_) are mainly due to NE-SW compression and then they were superimposed by a later brittle deformation of normal faults (D_3_). Fowler et al.^57^ defined an earlier deformation phase D_1_ evidenced by foliation (S_1_), asymmetric folds (F_1_), stretching lineations and pencil structures (L_1_) in the different ophiolitic associations. F_1_ folds and other lineations trend in N to NE and S to SW and plunges in different directions. This phase deformed later by D_2_ phase which evidenced by a NW-SE trending F_2_ fold that has been affected by E-W to ENE-WSW trending sinistral faults. Farahat et al.^92^ mapped the contact between the ophiolitic mélange and the granitic rocks as a fault contact.

##  Materials and methods

### Remote sensing datasets

The current research integrated multispectral (Sentinel 2 and Planetscope) and hyperspectral (PRISMA) data with various aspects to introduce accurate lithological discrimination and outline the main structural characteristics of the studied terrain. **Sentinel 2** (S2) data were applied for comprehensive lithological discrimination. With its multispectral sensor, Sentinel 2 satellite provide high-level land surface monitoring with a sweep width of approximately 290 km and is thus used for several geological applications^4,13,94,95^ based on its spatial and spectral features (Table 1).

**Table 1:** Characteristics of Sentinel-2 Data.

Band	Spectral region	Central wavelength (µm)	Spatial resolution (m)
**1**	Ultra blue	0.443	60
**2**	Blue	0.490	10
**3**	Green	0.560	10
**4**	Red	0.665	10
**5**	VNIR	0.704	20
**6**	VNIR	0.740	20
**7**	VNIR	0.782	20
**8**	VNIR	0.842	10
**8a**	VNIR narrow	0.865	20
**9**	SWIR water vapor	0.945	60
**10**	SWIR cirrus	1.375	60
**11**	SWIR	1.610	20
**12**	SWIR	2.190	20

VNIR = visible near infrared, SWIR = short wave infrared, and TIR = thermal infrared

For better analysis, **PRISMA** (PRecursore IperSpettrale della Missione Applicativa) hyperspectral data was applied due to its spectral wealth. The Italian Space Agency launched PRISMA, a sun-synchronous hyperspectral sensor, in March 2019^96,97^. It offers 250 spectral channels covering a wavelength range of 0.4–2.5 μm. Employing a push broom sensor configuration, PRISMA achieves a spatial resolution of 30 m for hyperspectral bands and an impressive 5 m for panchromatic, encompassing a swath width of 30 Km. For detailed spectral analysis in geological applications, PRISMA offers comprehensive data within the VNIR (66 channels, 400–1100 nm) and SWIR (174 channels, 920–2500 nm) spectra. Its spectral precision, with a width of ≤ 14 nm and calibration accuracy of ± 0.1 nm, elevates its performance. Moreover, the signal-to-noise ratios surpass notable thresholds, exceeding 160, 100, and 240 for VNIR, SWIR, and Panchromatic channels respectively, coupled with a radiometric quantization capability of 12 bits.

**Fig. 2:**
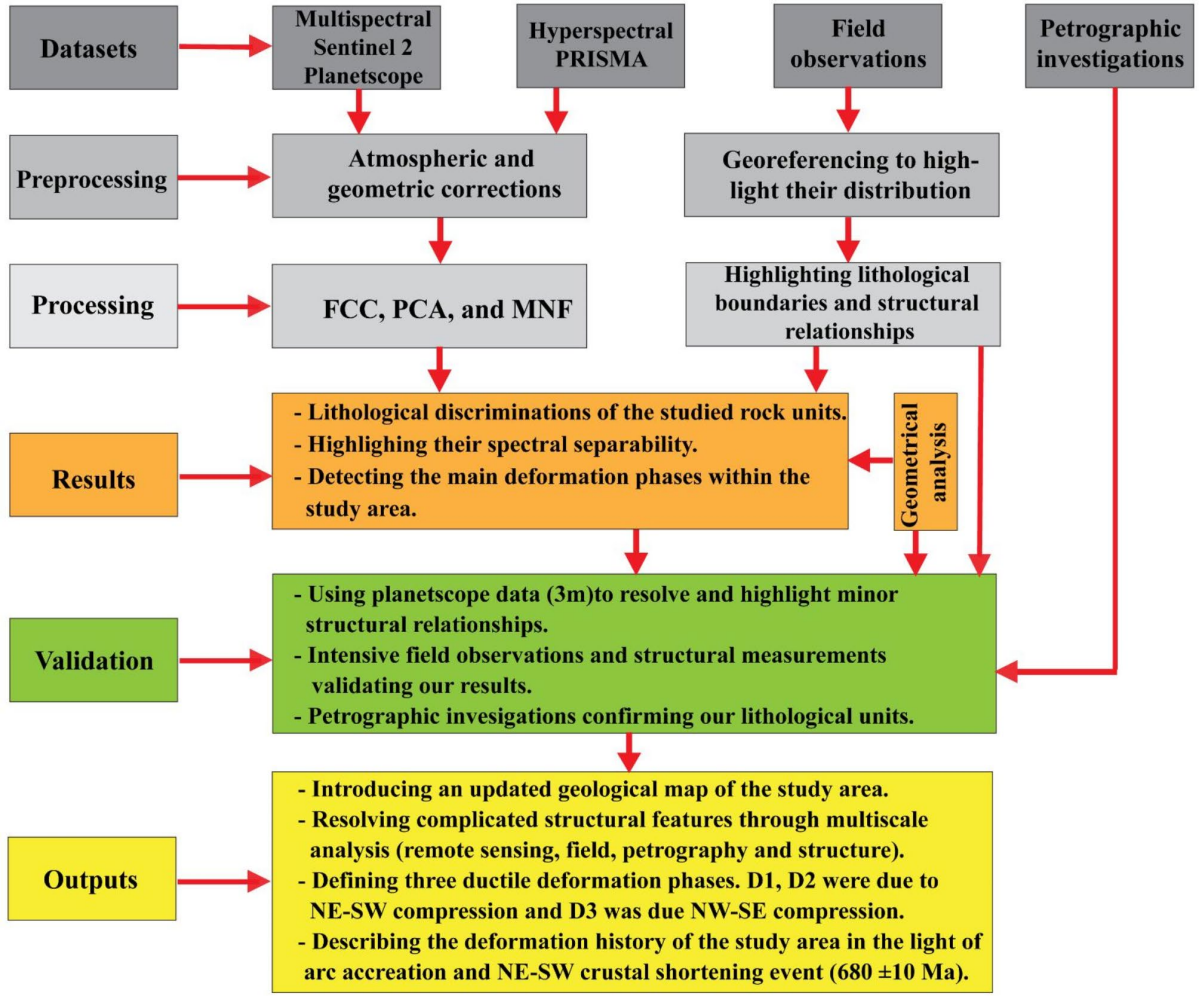
Flowchart illustrating the materials utilized and the methodology applied in this study.

Besides Sentinel 2 and PRISMA, our research employed the very high-resolution **planet scope** (3-meter pixel size) data to be able to detect several structural features and help resolving the structural setting of the studied terrain. The constellation of planetscope satellites (DOVEs), operating in a sun-synchronous orbit between 475 and 525 km above Earth^98^. It is a daily repeating constellation whose equatorial passes usually occur between 9:30 and 11:30 am local solar time. The Planetscope DOVE cubesat provides a suite of eight bands for our investigation: red edge, red, green, green I, yellow, blue, coastal blue, and near-infrared. Beside these datasets, all of the available geological, tectonic, structural and geochronological information were gathered and thoroughly examined, besides, maps (e.g. geologic map of Egypt of scale 1: 2,000,000^99^ were utilized for the preliminary geological interpretation of the present area. The whole methodology adopted in the current research are presented in a flowchart as shown in Fig. 2.

### Methods

The widely-approved image processing methods including false color composites (FCC), principal component analysis (PCA), independent component analysis (ICA), minimum noise fraction transform (MNF) are applied to the various datasets. Due to its usefulness in geological applications, SWIR bands are widely approved to uncover lithological characteristics. Thus, a main FCC applied in our research is S2 12-11-2 in RGB helping in general lithological differentiation^78,100^. Principal Component Analysis (PCA) stands as a recognized technique for reducing dimensionality, with the initial principal components capturing the utmost variability within the data^12,101^ making them pivotal for constructing robust color composites. So, we utilized these combinations for enhancing the main lithological boundaries. ICA represents a specific instantiation of blind source separation (BSS), tasked with disentangling source signals from mixed signals absent substantial prior knowledge of the sources or the mixing process^102–104^. Thus, the core aim of ICA lies in identifying a set of uncorrelated components that strive for maximal independence from each other^105^. Unlike other components (e.g. PCA), which are ordered in descending order of variance, MNF encompasses an orthogonal rotation process designed to yield components arranged in ascending order of random noise magnitude^13,106,107^. MNF typically entails a dual-phase procedure involving PCA rotations: firstly, discerning the principal components of the noise covariance matrix to decorrelate and rescale data noise, and secondly, extracting principal components from the noise-whitened data.

High resolution natural color satellite images (1 m/pixel resolution) are produced, registered and enhanced for further detailed analysis. These images are used for the detection of additional details (foliation orientations, axes of major folds and fault traces). The previously mentioned data are used as successive layers in the GIS database together with our field observations to produce the final geologic map of the study area.

Field work was carried out by following well-organized computer-based real-time tracking routes using ETM + images, Google Earth images and previously constructed maps as layers on the Global Mapper software (v. 15), which were used in the field navigation aided by a GPS device. Field work has been carried out through about 95 selected stations along definite traverses and comprised sample collections, measurements for different structural elements.

Following image processing techniques and fieldwork, a detailed petrographic examination was conducted for the prepared thin sections, under polarizing microscope (Optikia.B-150POL-M). The microscopic study includes petrographic and mineralogical characteristics of the different rock types, abundances and types of phenocrysts in volcanic rocks, volumetric proportion and mineralogy of groundmass, and the individual textural relations exist between mineral grains. Photomicrographs portraying the different rock varieties and characteristic features were taken using polarizing microscope attached camera (Nikon) at the Geology Department, Faculty of Science, Cairo University, Egypt. The structural data are geometrically treated using the stereographic projection techniques based on technique proposed by^108^ for the areas, which were affected by multiple phases of deformation. The lower hemisphere Schmidt equal area projection is used in the present analysis.

##  Results

### Remote sensing findings

**Fig. 3:**
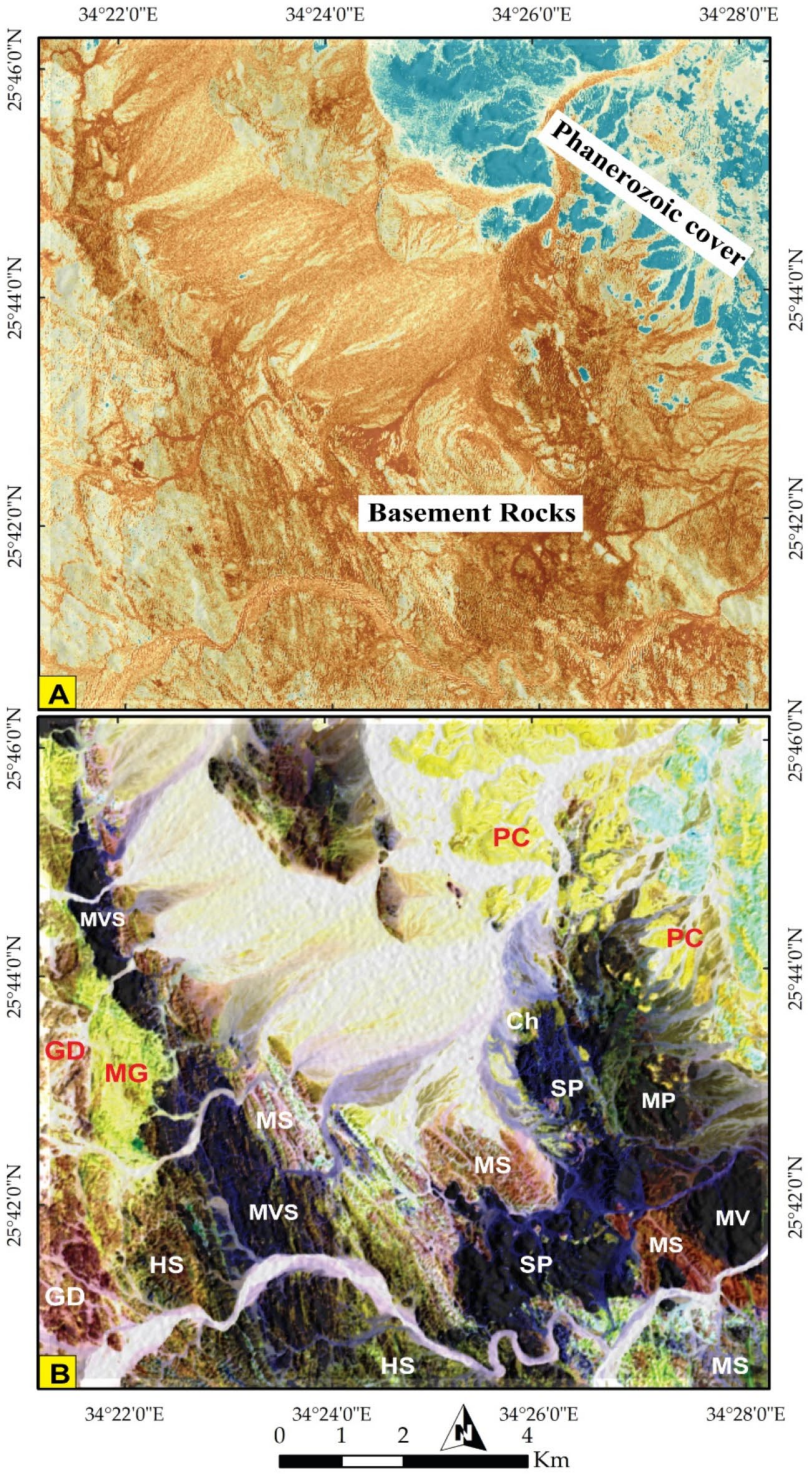
**(A)** General discrimination of Phanerozoic rocks (highlighted in cyan blue at the NE corner) from the basement rock units using Sentinel 2 pseudocolor map of IC 3 and **(B)** Lithological differentiation using Sentinel 2 FCC 12-6-2 in RGB. (SP) Serpentinite, (MP) Metapyroxinite, (MS) Metasediments, (MVS) Metavolcano-sedimentary rocks, (MV) Metavolcanics, (HS) Hornblende Schist, (MG) Monzogranite, (GD) Granodiorite, and (PC) Phanerozoic cover. The figure was created by ENVI v. 5.6.2. software; (https://www.l3harrisgeospatial.com/Software-Technology/ENVI*)*

The various sensors utilized provided multiscale observations, enabling both broad and detailed insights that aided in delineating the geological characteristics of the study area. To primarily discriminate lithological formations, S2 IC7 (Fig. 3A) was employed to differentiate the Phanerozoic sedimentary cover (PC) in cyan blue from the intricate basement rocks (brown). To untangle the complexity within the basement rocks, Sentinel 2 FCC 12-6-2 in RGB yielded discernible results (Fig. 3B), facilitating the differentiation of ophiolitic serpentinite (SP) blocks (depicted in black) and highlighting Miocene rocks and Quaternary deposits. The mélange matrix, primarily composed of metasediments (MS), was visualized in reddish-brown. The island arc assemblage (Metavolcano- sedimentary rocks (MVS), Metavolcanics (MV), and Hornblende schist (HS)) was denoted by dark blue, while pinkish and bright yellow colors were assigned to granodiorite (GD) and monzogranitic rocks (MG), respectively.

**Fig. 4:**
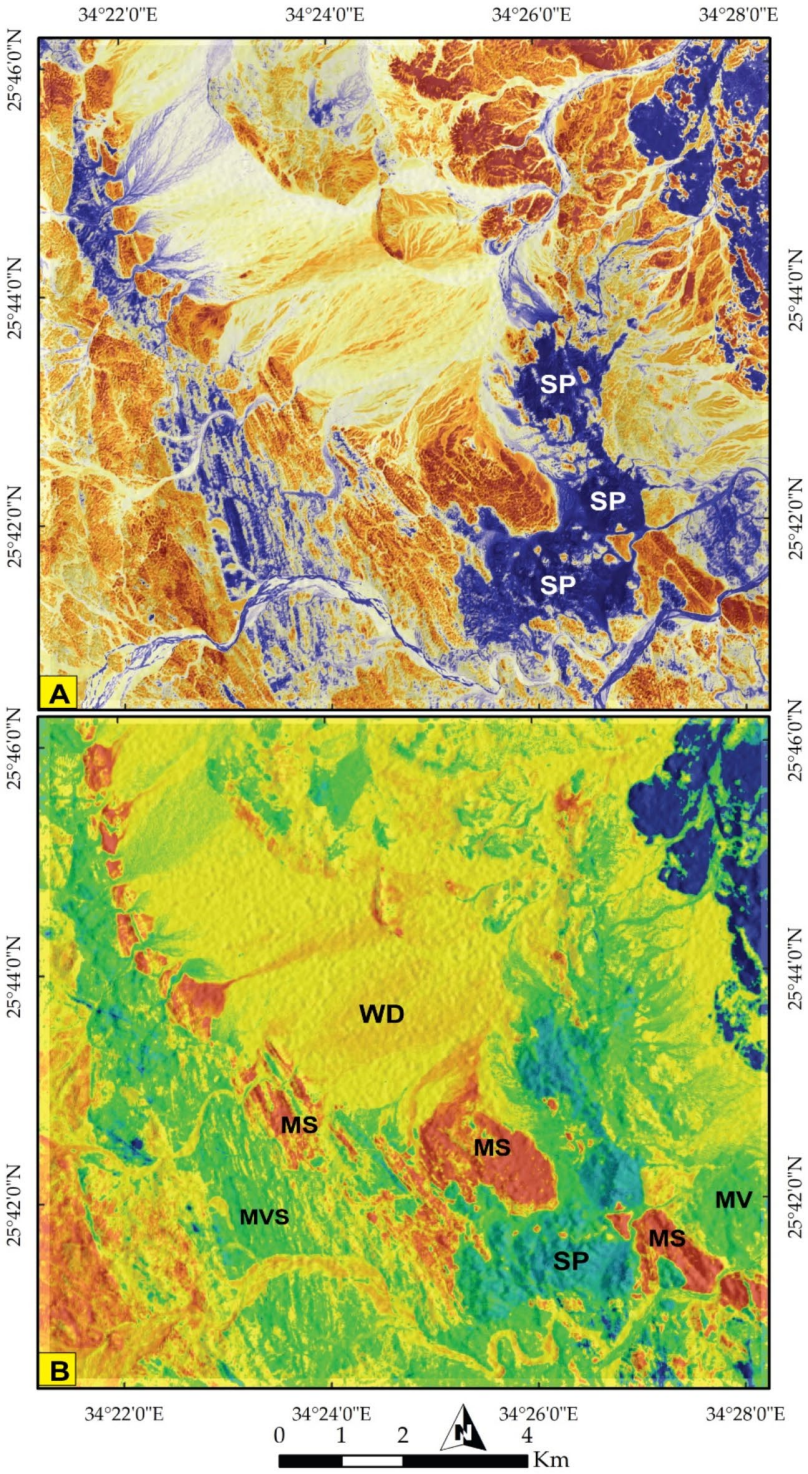
Lithological discrimination using Pseudocolor maps of Sentinel 2 data **(A)** PC2 clearly highlighting serpentinites in a deep blue color and **(B)** MNF 2 discriminating serpentinites (greenish blue) from its mélange matrix (red) and island arc assemblage (green). (SP) Serpentinite, (MS)Metasediments, (MVS) Metavolcano-sedimentary rocks, (MV) Metavolcanics, (WD) Wadi Deposits. The figure was created by ENVI v. 5.6.2. software; (https://www.l3harrisgeospatial.com/Software-Technology/ENVI*)*

To further delineate these rock units, specific combinations were employed to discriminate the main lithological boundaries. For instance, the colored representation of S2 PC2 highlighted the boundary of SP in an intense blue color, clearly distinguished from other rock units (Fig. 4A). This distinction was corroborated using S2 MNF2, where SP appears as cyan blue, distinctly separated from MS (in red and blue), island arc assemblages (in green), and wadi deposits (in yellow) (Fig. 4B).

**Fig. 5:**
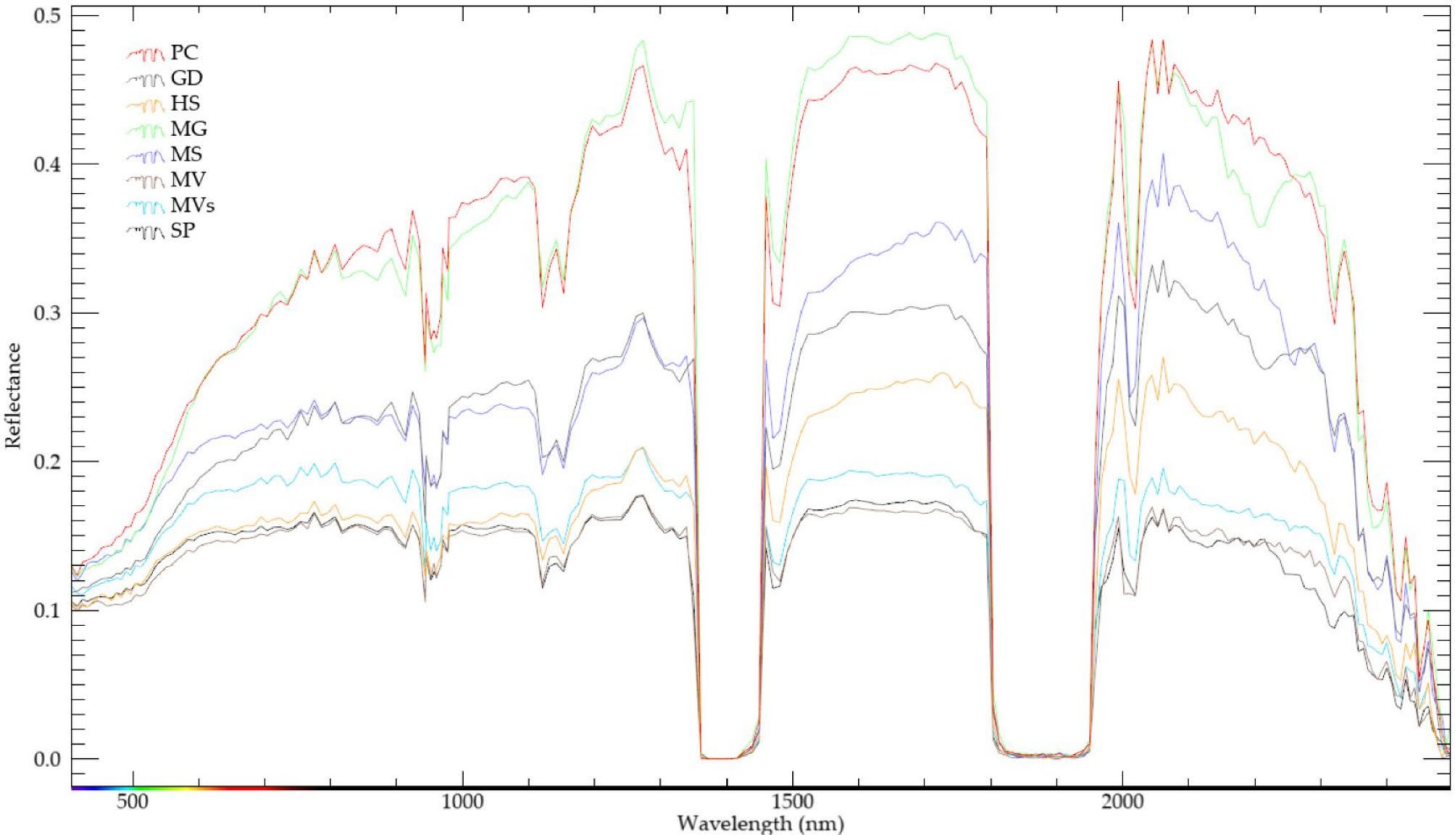
Spectral differentiation of the lithological units using PRISMA data. (SP) Serpentinite, (MS) Metasediments, (MVS) Metavolcano-sedimentary assemblage, (MV) Metavolcanics, (HS) Hornblende Schist, (MG) Monzogranite, (GD) Granodiorite, and (PC) Phanerozoic cover.

**Fig. 6:**
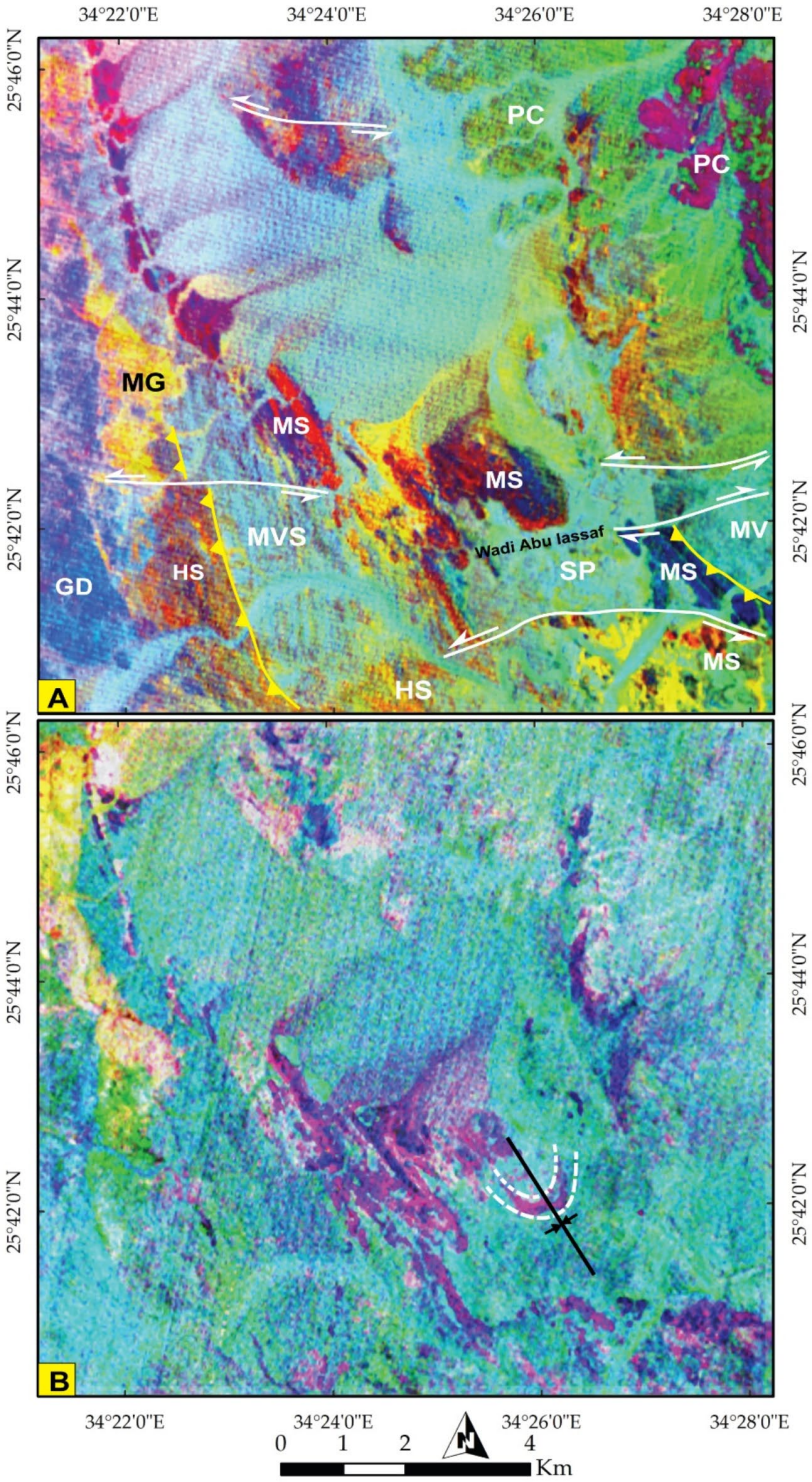
Lithological discrimination using PRISMA hyperspectral images of **(A)** PC4-PC5-PC6 in RGB and **(B)** Intensive folding (purple-colored rocks) introduced through IC7-IC8-IC9 in RGB respectively. (SP) Serpentinite, (MS) Metasediments, (MVS) Metavolcano-sedimentary assemblage, (MV) Metavolcanics, (HS) Hornblende Schist, (MG) Monzogranite, (GD) Granodiorite, (PC) Phanerozoic cover. The figure was created by ENVI v. 5.6.2. software; (https://www.l3harrisgeospatial.com/Software-Technology/ENVI*).*

In order to enhance spectral differentiation among these rock units (Fig. 5), PRISMA combinations were utilized, despite its lower spatial resolution compared to S2 images. PRISMA PC4-PC5-PC6 in RGB respectively highlighted the main lithological units. In Fig. 6A, Miocene rocks (Phanerozoic cover) and terraces are depicted in shades of purple and light green, arc assemblage metavolcanics in solid blue, massive and sheared serpentinites in cyan, MS in red, MG in bright yellow, HS (just below the monzogranite) in a brownish hue, and GD in bright blue.

**Fig. 7:**
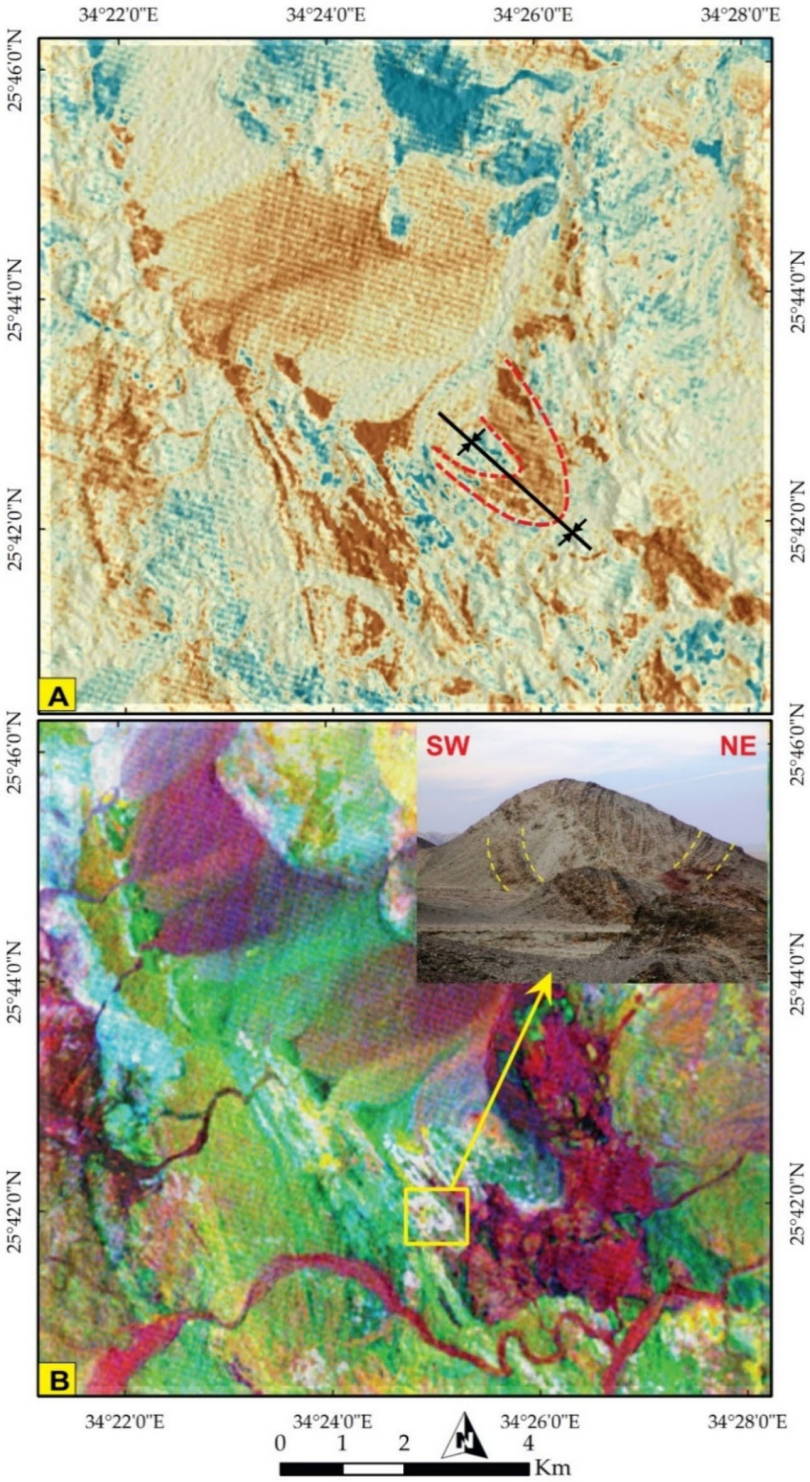
Manifestation of the highly deformed rocks (brown) with notable depiction of folds using PRISMA data **(A)** Pseudocolor map of PC 12 and **(B)** PC12-MNF8-IC7 in RGB respectively. The inset in figure **B** showing a mesoscopic syncline identified from deflection of foliation directions. The figure was created by ENVI v. 5.6.2. software; (https://www.l3harrisgeospatial.com/Software-Technology/ENVI*).*

**Fig. 8:**
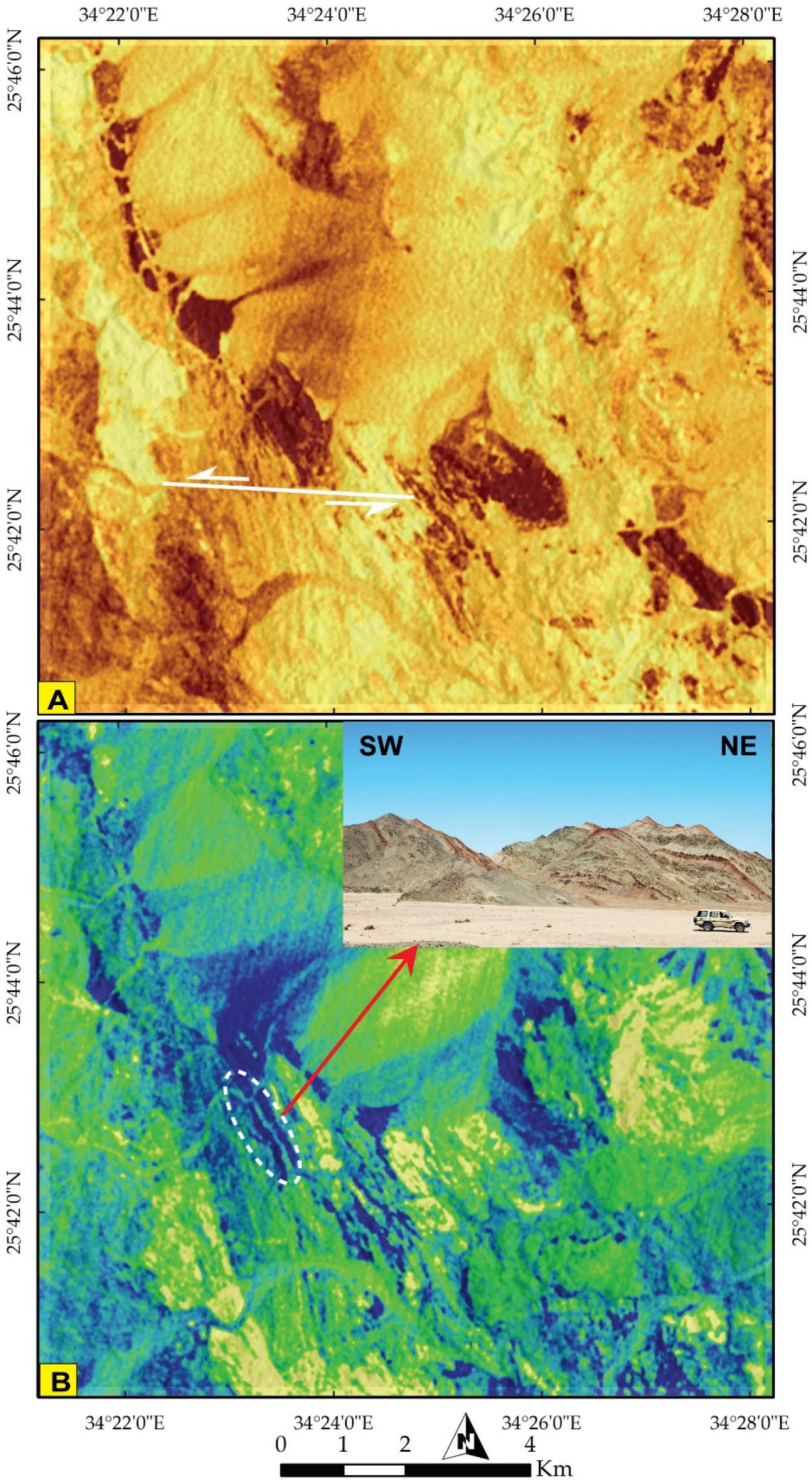
**(A)** Clear depiction of the effect of the sinistral E-W trending strike-slip faults through the displacement of metasediments (brown), and **(B)** highlighting the main structural elements trending NNW (blue lines) within the ophiolitic melange matrix and island arc assemblage. The inset in Figure **B** showing the main foliation direction. The Images were obtained from ISA and the figure was created by ENVI v. 5.6.2. software; (https://www.l3harrisgeospatial.com/Software-Technology/ENVI*).*

Through IC7-IC8-IC9 in RGB, conspicuous folding was manifested at the central part of the map (Fig. 6B). These folding and highly deformed zones were visualized by a pseudocolor map of PC 12 (Fig. 7A), showcasing the discontinuities of several lithological bodies (in brown) and a clear depiction of folding (Fig. 7B) by tracking the configuration of bright white pixels of PRISMA PC12-MNF8-IC7 in RGB, respectively. This folding is confirmed from ground truth (inset in Fig. 7B). As one of the core aims of the current research is deciphering the structural setting, a special emphasis was given to highlight such discontinuities that highlight predominantly the effect of strike slip displacements by investigation the configuration of brown blocks in Fig. 8A. Additionally the folding regime and one of the common structural features (NNW trending foliation) were well-depicted in Fig. 8B by following the blue and yellow pixels in the central part of the map, which is also well-noticed in field (inset in Fig. 8B).

**Fig. 9:**
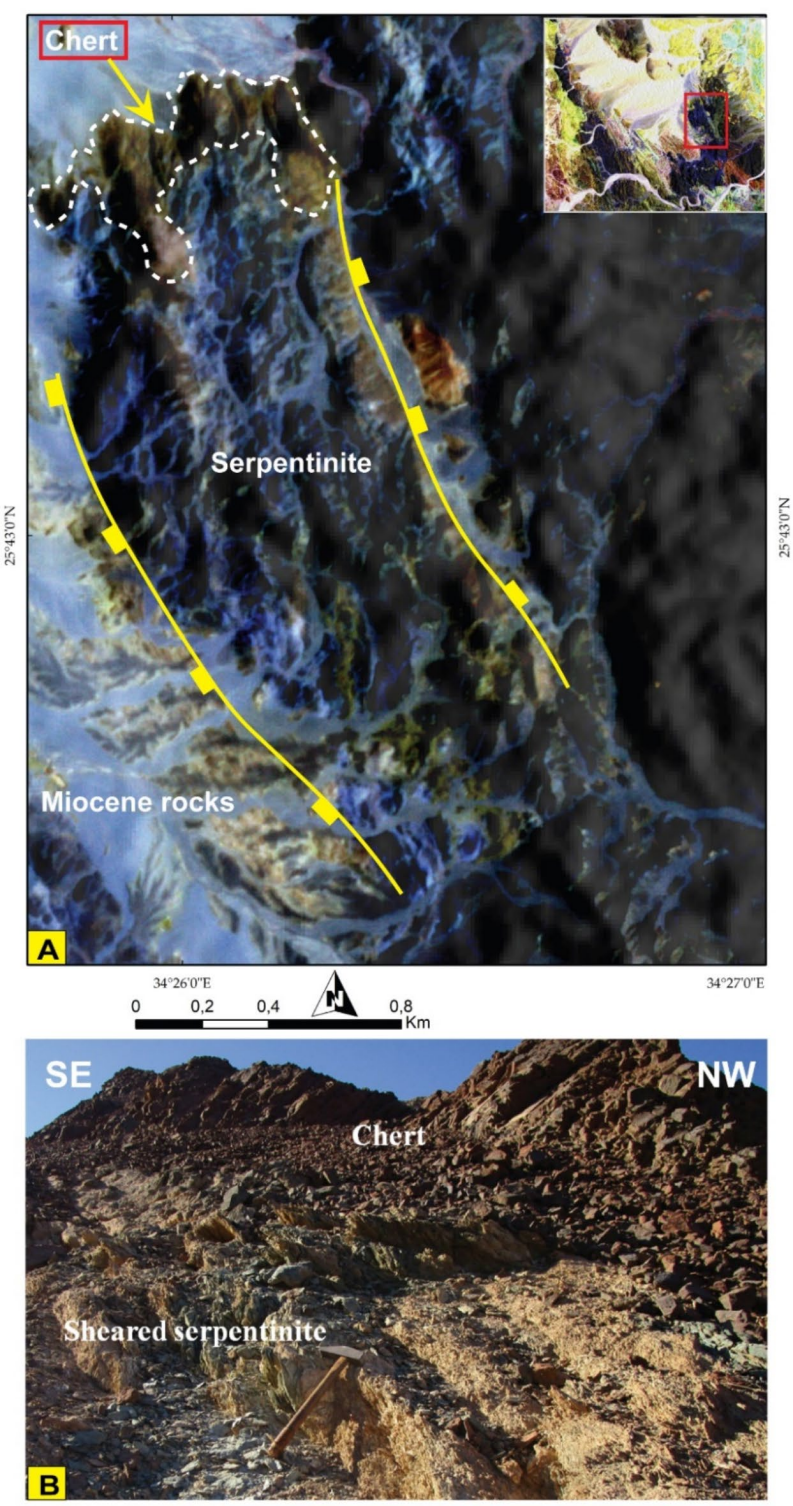
**(A)** A 3 m Pixel size planet scope FCC of 8-6-2 bands in RGB highlighting the smallest rock unit of the studied area (chert) as small exposure to the north of serpentinite rocks. **(B)** Field photograph showing chert ridge rest over sheared serpentinite in the northeastern part of the study area. The Image were obtained from Planet.com and the figure was created by ENVI v. 5.6.2. software; (https://www.l3harrisgeospatial.com/Software-Technology/ENVI*).*

**Fig. 10:**
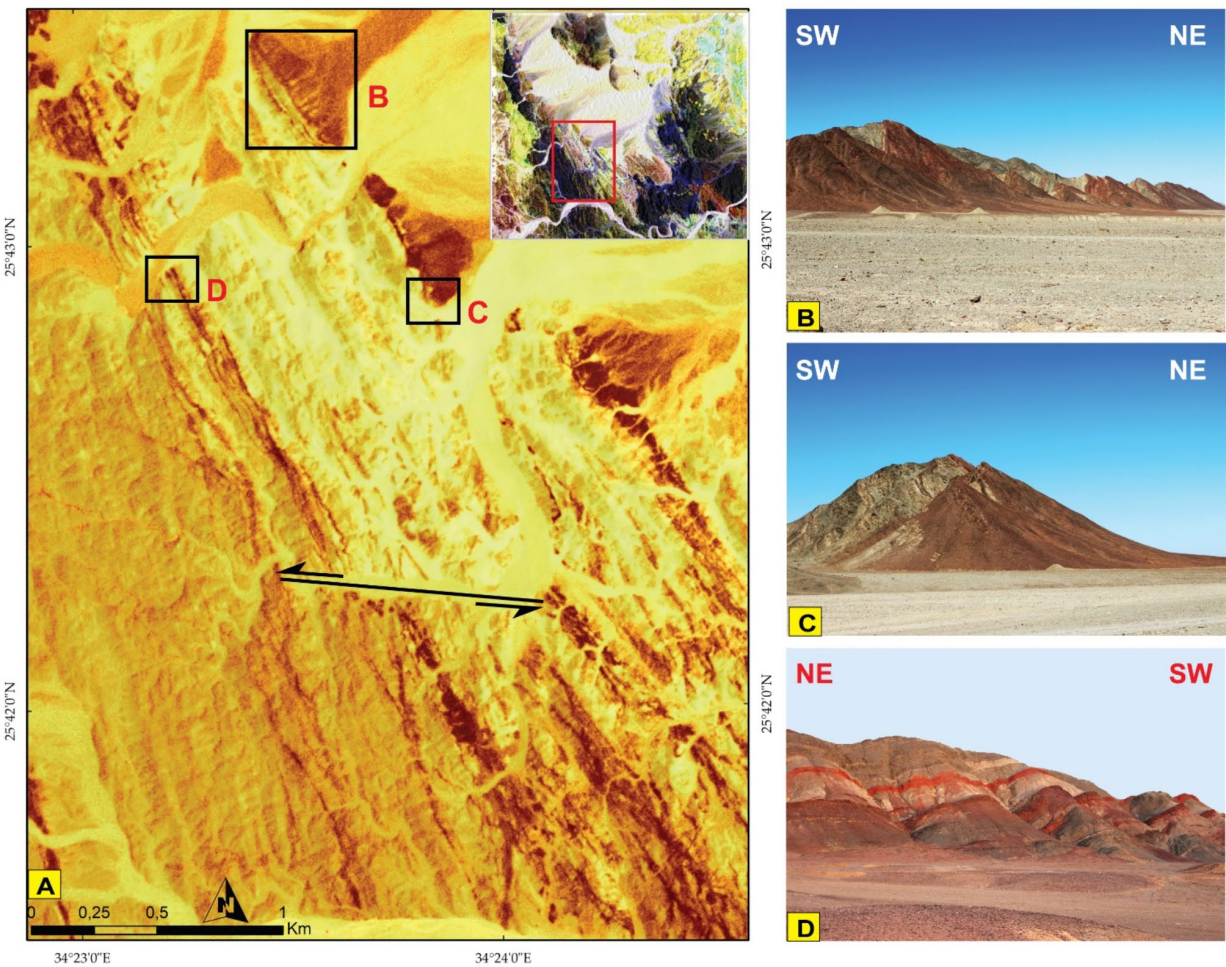
**(A)** A 3 m Pixel size planet scope pseudocolor map of MNF 2 highlight in the main structural elements. Note the sinistral strike slip displacement is clear at the central part. (**B**, **C**, and **D**) field photographs for acidic dyke swarms cut through MS. Black boxes over figure A shows the exact locations of figures B, C, and D. The Image were obtained from Planet.com and the figure was created by ENVI v. 5.6.2. software; (https://www.l3harrisgeospatial.com/Software-Technology/ENVI*)*

PlanetScope data corroborated our findings, and thanks to its very high spatial resolution (VHR), it was utilized for detecting minor lithological units. For instance, the distribution of chert as an associated rock unit with serpentinite was clearly depicted, enabling precise separation and mapping of the boundary between them, as illustrated in Fig. 9A of the Planet FCC of 862 in RGB and supported by field investigations (Fig. 9B). In addition, planet scope pseudocolor map of MNF 2 clearly discriminate acidic dyke swarms dissected through MS (Fig. 10A) which is supported by high quality field photographs (Fig. 10B, C, D).

**Fig. 11:**
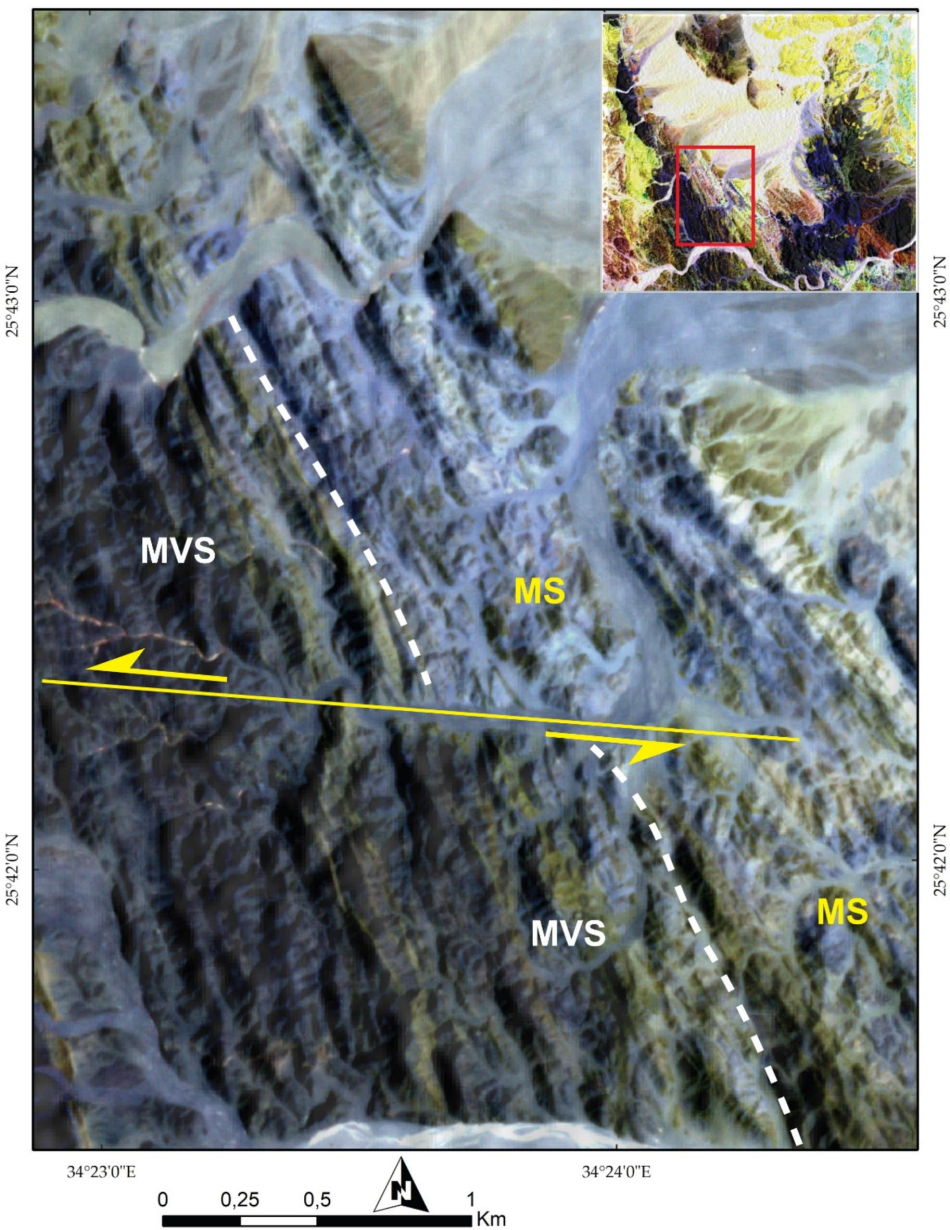
A 3 m Pixel size planet scope FCC of 862 in RGB respectively clearly highlighting the NNW trending tectonic fabric with a prominent manifestation of E-W trending sinistral strike slip fault. (MS) Metasediments, (MVS) Metavolcano-sedimentary assemblage. The Image were obtained from Planet.com and the figure was created by ENVI v. 5.6.2. software; (https://www.l3harrisgeospatial.com/Software-Technology/ENVI*).*

**Fig. 12:**
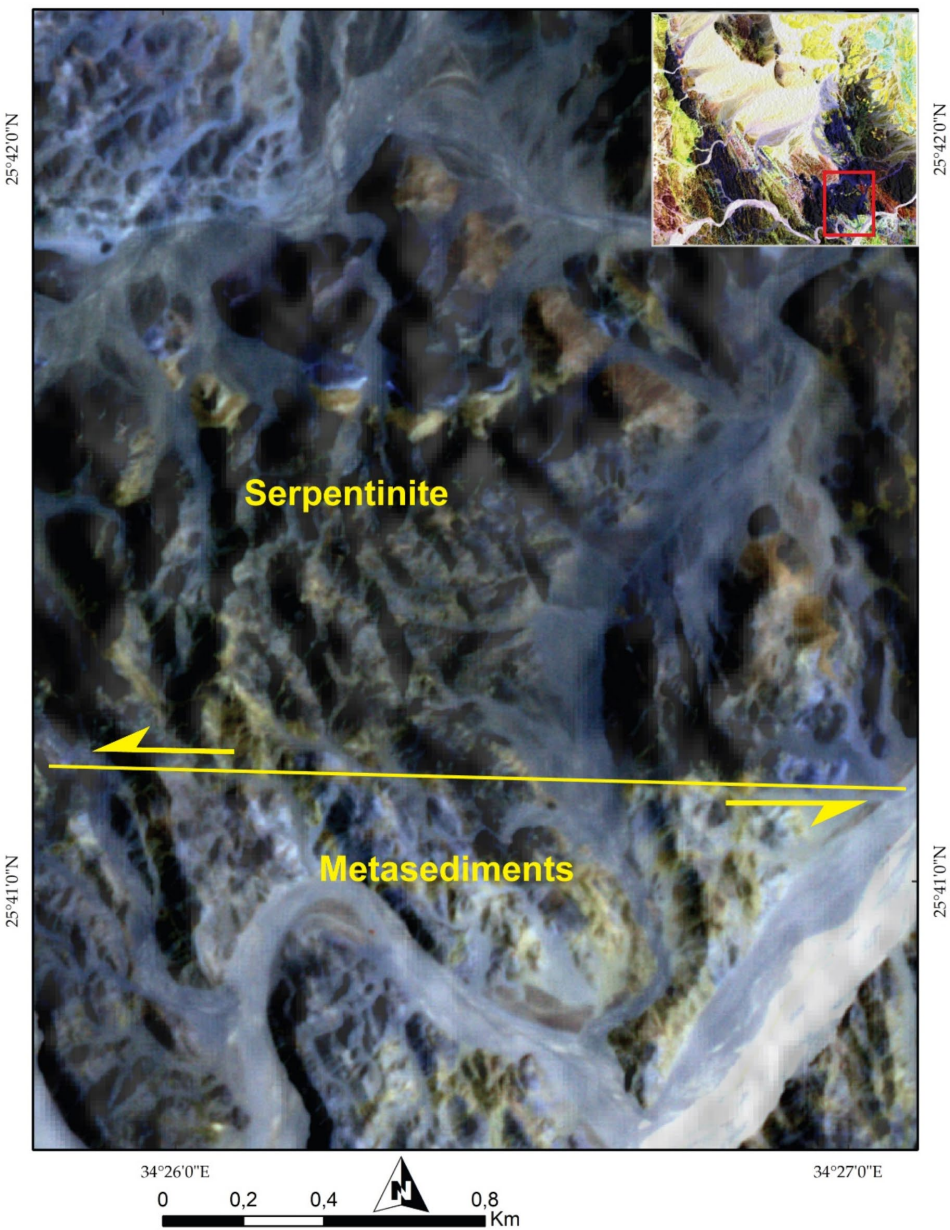
A clear demarcation of an E-W strike-slip fault is evident, distinguished by the tonal contrast between the dark upper part (serpentinites) and light lower part (metasediments) layers, depicted using Planet data FCC of 862 in RGB, respectively. The Image were obtained from Planet.com and the figure was created by ENVI v. 5.6.2. software; (https://www.l3harrisgeospatial.com/Software-Technology/ENVI)

**Fig. 13:**
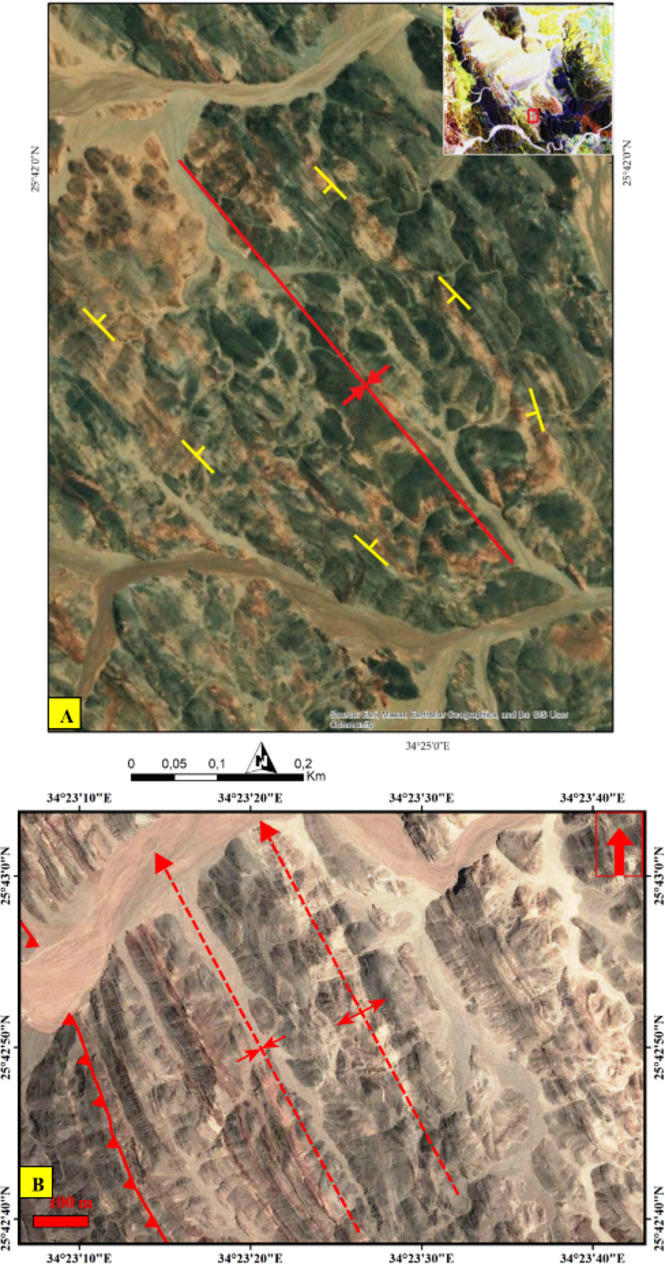
**(A)** A clear demarcation of deflection of foliation dipping that clarified a major syncline, depicted using Planet data FCC of 862 in RGB, respectively. **(B)** Natural colour high resolution satellite image extracted from Google Earth showing inversion in dip direction of foliation, resulting in (F_2_) anticlinal and synclinal folds in the western parts of the study area close to the thrust contact between the metasediments and the metavolcano-sedimentary unit. The Image were obtained from Planet.com and google Earth, and the figure was created by ENVI v. 5.6.2.software; (https://www.l3harrisgeospatial.com/Software-Technology/ENVI*).*

**Fig. 14:**
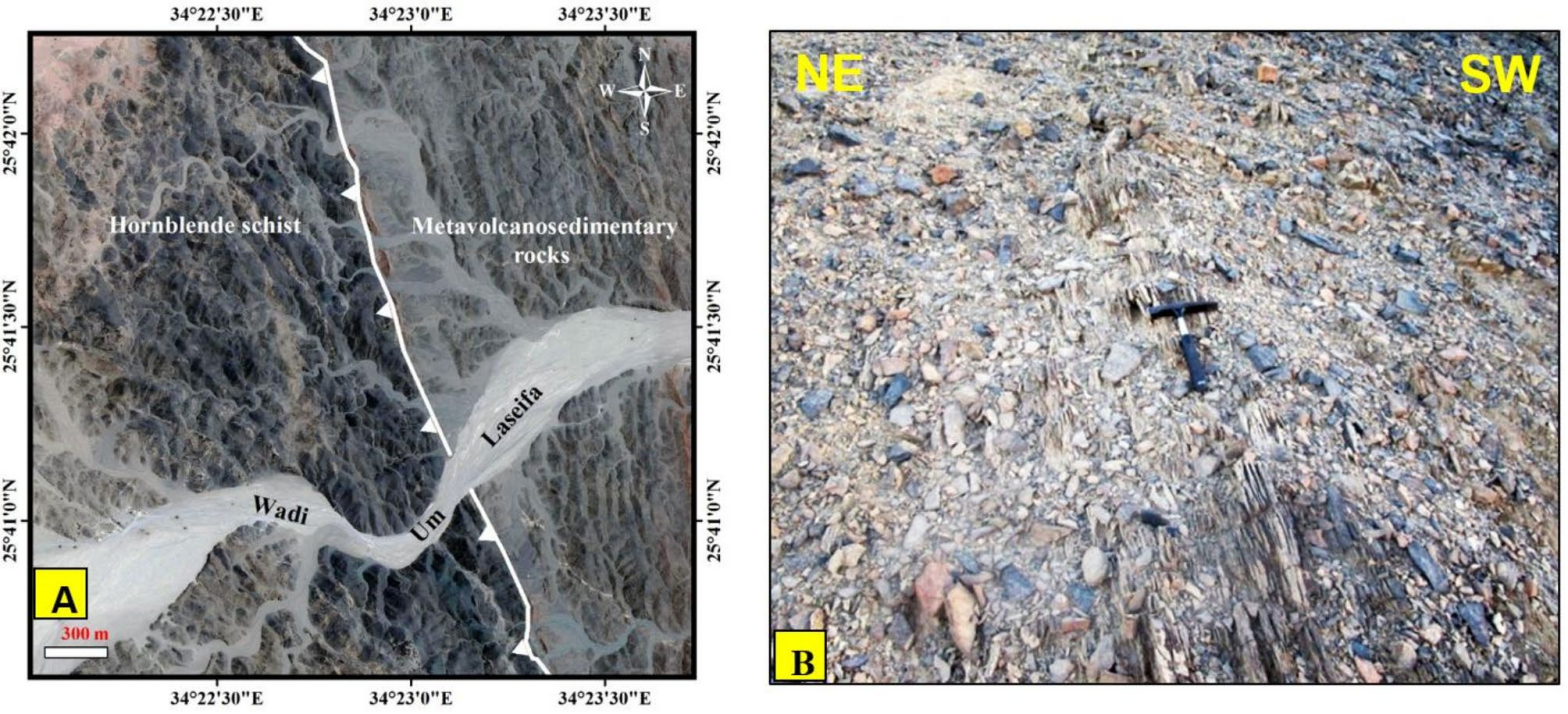
**(A)** Natural colour landsat high resolution satellite image extracted from Google Earth showing the major thrust contact between metavolcano-sedimentary and hornblende schist belts at the SW parts of the study area. **(B)** Mylonitic foliation along thrust contact in Fig. 12A, Wadi Um Laseifa. The Image were obtained from google Earth, and the figure was created by SmartSketch v. 4.0 software; https://smartsketch.software.informer.com/4.0/.

To fully exploit such VHR data, numerous structural features were meticulously examined, aiding in the delineation of the main structural regime within the studied terrain. For example, the PlanetScope FCC of 8-6-2 in RGB (Figs. 11 and 12) distinctly highlighted the left-lateral strike-slip faulting visualized in the central and southeastern part of the study area using the same spectral combination that clarified displacements of these blocks and facilitating accurate structural mapping. Another regime featuring folding, thrusting. Additionally, over a metric scale (e.g., 50 m), common deformational regimes such as folding were detected, as depicted in Fig. 13A and supported high resolution Google Earth image (Fig. 13B). Also, Google Earth images clearly define thrusting deformation regime (Fig. 14A).

### Field and petrographic investigations

Remote sensing data used in lithological discrimination and structural deciphering have been confirmed through extensive field, petrographic and structural studies. This detailed analysis confirms the previously mentioned remote sensing findings and help integrating and completing the whole lithological sequence within the study area. Based on our remote sensing findings, and petrographic field work, our study highlighted the following rock units as the main lithological constituents:

#### Ophiolitic mélange association

- *Serpentinites (SP)*.
  Our field observations confirmed the remote sensing results (Figs. 3B and 4B, and 9) and revealed that serpentinites occupy the trough of the major syncline as conspicuous mountainous ridges in the central sector and extend along its eastern limb, in addition to their presence as thrust sheets of limited extension along minor thrusts trending NW-SE (Fig. 15A). They are separated from the central west by Miocene sandstone and delimited from the east by meta-pyroxenites (Fig. 3). They show varieties of massive and sheared serpentinites (Fig. 15B), talc carbonates, listwanite and include relicts of the fresh dunite, prediotite and pyroxenite. Microscopically, SP consist of antigorite, chrysotile and bastite associated with carbonates, magnetite and chromite (Fig. 16A). The dominancy of antigorite supports the idea that these rocks represent ocean floor metamorphism^109^. Talc carbonates are dominant by sheared talc, carbonate minerals and serpentine relics (Fig. 16B), whereas listwanite differs from it by richness of quartz.
  **Fig. 15:**
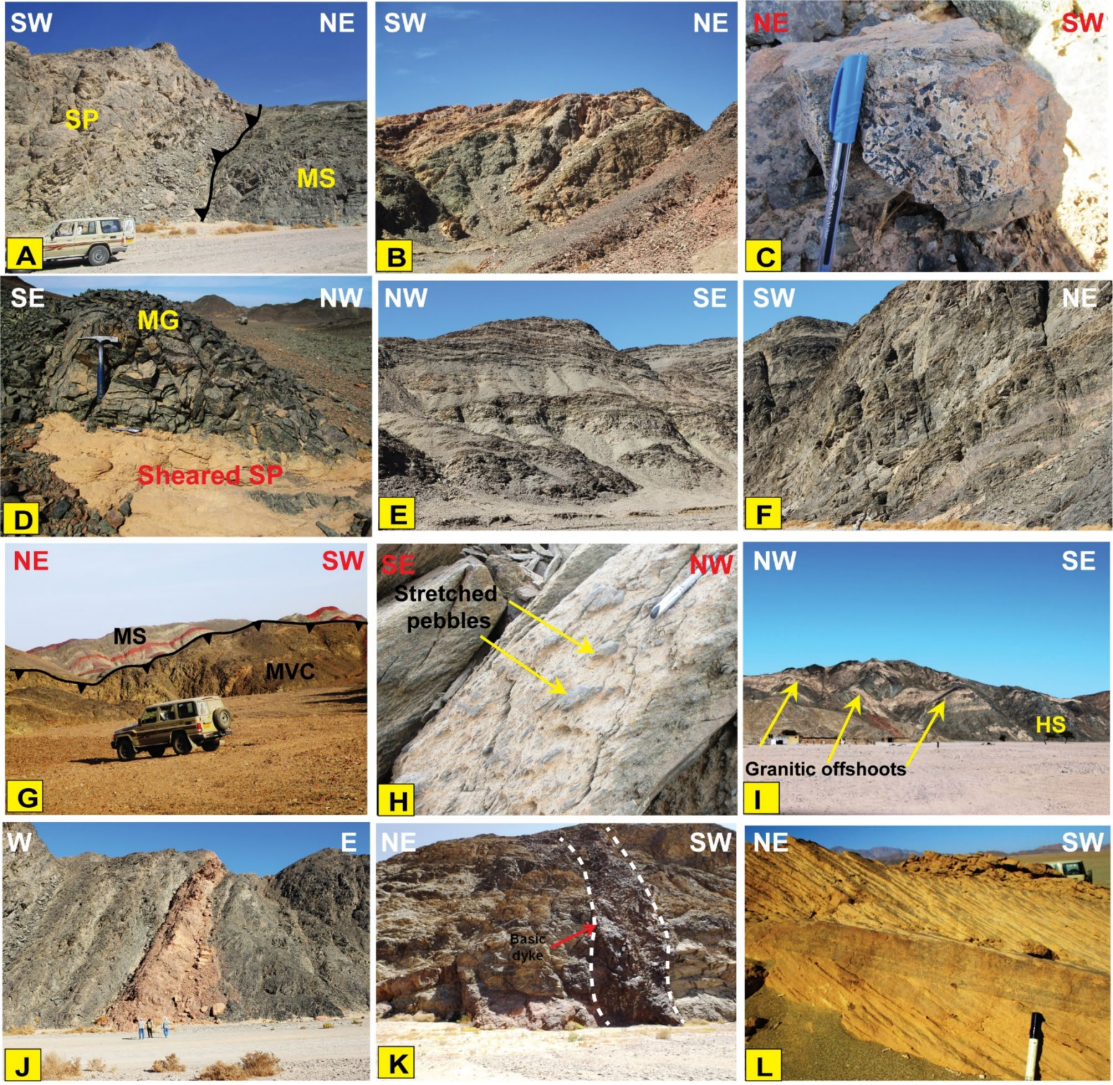
Field photographs showing **(A)** Sheets of serpentinites thrust over metasediments, Wadi Um Gheig. **(B)** Sheared serpentinite block, central sector, north of Wadi Um Laseifa. **(C)** Prismatic crystals of hornblende in metapyroxenite rocks in the NE of the study area. **(D)** Metagabbro (MG on the figure) thrusted over serpentinite in the central sector of the study area. **(E)** Well-developed foliation in the metasediments along Wadi Um Laseifa. **(F)** Well-developed foliation in the metavolcanics at the entrance of Wadi Um Gheig. **(G)** The thrust contact between the MVS rocks and the MS at the NW of the study area. **(H)** Stretched pebbles in the MVS assemblage. **(I)** Granitic offshoots intruding the hornblende schist at the extreme SW of Wadi Um Laseifa. **(J)** Acidic dyke intruding the metavolcanics at the entrance of Wadi Um Gheig. **(K)** Basic dyke cutting through the metavolcanoclastics, Wadi Um Laseifa. **(L)** Miocene sandstone hills with low relief, Wadi Wizr. (Created by SmartSketch v. 4.0 software; https://smartsketch.software.informer.com/4.0/)
  **Fig. 16:**
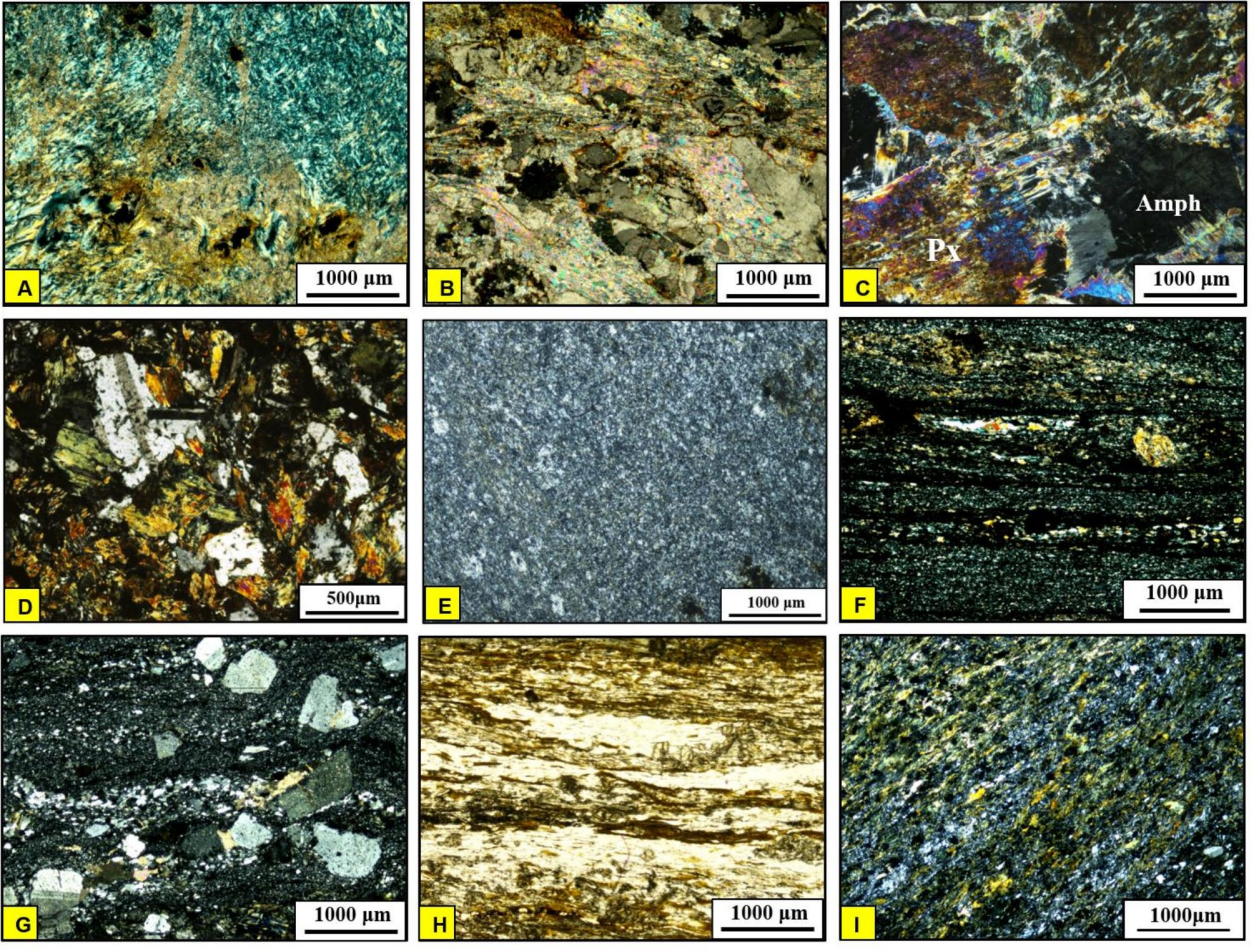
Photomicrograph showing **(A)** Calcite patches and veins in serpentinite (CN); **(B)** Talc shreds and fibers forming dense fine-grained groundmass in the talc carbonate rocks (CN); **(C)** Coarse-grained pyroxene and amphibole crystals forming hypidiomorphic texture (CN); **(D)** Hornblende and plagioclase intergrowth forming subophitic texture (CN); **(E)** Microcrystalline quartz aggregates in chert (CN). **(F)** Alternating light and dark bands (CN); **(G)** Graded bedding represented by fining of quartz and feldspars upwards (CN); **(H)** Primary lamination in biotite schist (PPL); **(I)** Mylonitized meta-andesite with foliated matrix formed of hornblende, plagioclase (CN). (Created by SmartSketch v. 4.0 software; https://smartsketch.software.informer.com/4.0/*)*

- *Metapyroxenites (MP)*.
  Metapyroxenite is observed for the first time in the study area (Fig. 3B). It is exposed along the eastern sector, bordered from west by serpentinite, from east by terraces and Miocene sediments, juxtaposed from the north against metavolcanics along thrust fault and from the south against metavolcanics and metasediments along E-W strike slip fault. It is characterized by the presence macroscopic prismatic crystals of hornblende (Fig. 15C), and microscopically formed of hornblende, actinolite, tremolite, chlorite, and primary augite relics (Fig. 16C).

- *Metagabbro*.
  This rock unit occurs as small unmappable dispersed masses in the ophiolitic matrix with black colours, yellowish brown weathered surfaces and exhibit fine- to medium-grained texture in different forms (Fig. 15D). Plagioclase and hornblende are essentially made up the metagabbro (Fig. 16D). Metagabbro in the present area are comparable with the “Older Metagabbro” in Egypt, observed elsewhere and described by many authors^79,110–114^.

- *Chert (Ch)*.
  Chert occurrence is limited to the eastern part of Wadi Wizr as a mappable block in contact with the serpentinite (Fig. 9). It is mostly composed of microcrystalline quartz with trace amounts of opaque minerals (Fig. 16E).

- *The Metasediments (MS)*.
  A well foliated and intensively deformed metasedimentary rocks, (Fig. 15E) occupy the central part surrounding the SP almost from all directions and extend from Wadi Um Gheig and Wadi Um Laseifa in the south to northwest corner in a NW direction (Figs. 3B and 4B). These rocks are bounded from east and west by MV and MVS respectively along a thrust contact (Fig. 15G). MS are represented by fine-grained and different colours varieties of metamudstones, metagreywackes, biotite and chlorite schist. They are pelitic to psammetic and made up of alternating light bands (quartz, calcite and k-feldspar) and dark bands (chlorite, sericite and clay minerals) (Fig. 16F). Metagreywackes mainly formed of crystal fragments of plagioclase feldspar, quartz and k-feldspar and epidote embedded in a foliated matrix of chlorite, sericite, muscovite and quartz and showing a characteristic graded bedding (Fig. 16G). Biotite schist consists mainly of biotite and quartz (Fig. 16H). Chlorite schist consists of chlorite, calcite, quartz and feldspars.

#### Arc assemblage

The arc assemblage is intensely deformed as they have mineral phases pointing out to medium or high-grade metamorphism (biotite ± hornblende, and garnet) and comprises metavolcanics, metavolcano-sedimentary assemblage and hornblende schist.

i. *Metavolcanics (MV)*.
  MV represented by metabasalts, meta-andesites, metatuffs and coarse-grained metapyroclastics varieties are exposed at the southeastern sector with intensive foliation (Fig. 15F) at the entrance of Wadi Um Gheig and at the northeastern part. Our field observations showed that it is bounded from the western and southern sides by both sheared serpentinite and metapyroxenite along thrust fault. Petrographically, MV comprise porphyritic varieties of metabasalt, meta-andesites and the latter is mylonitized along the thrust planes (Fig. 16I). The meta tuffs include welded tuffs, lapilli tuffs, and characterized by vesicular and amygdaloidal textures (Fig. 17A). Generally, the essential minerals made up MV are plagioclase, amphiboles, pyroxene set in a foliated matrix of same minerals, in addition to quartz.
  **Fig. 17:**
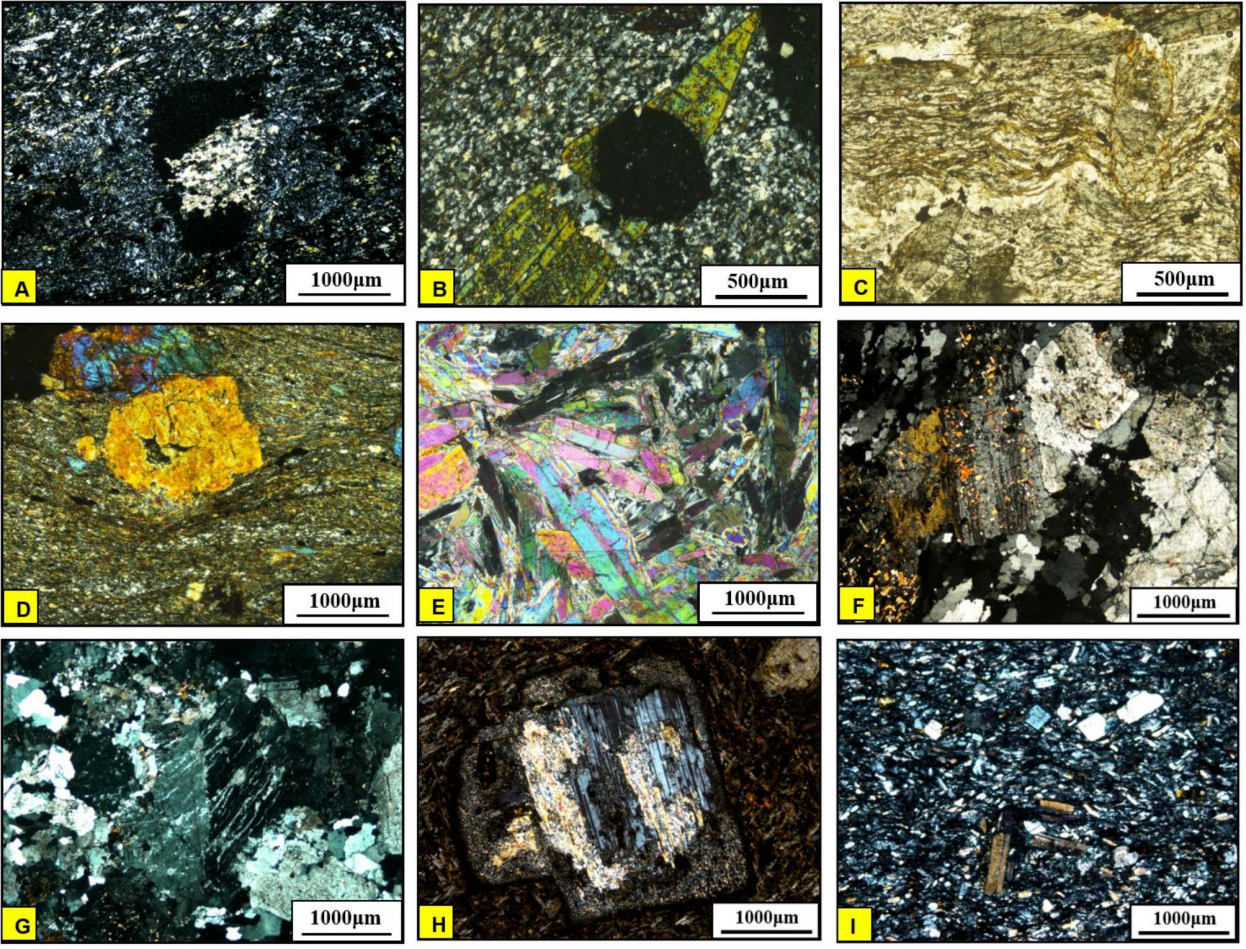
Photomicrograph showing **(A)** Meta-andesitic lapilli tuffs with amygdaloidal texture, the vesicles are partially filled with calcite (CN); **(B)** Actinolite crystal enclosing garnet indicating its post-tectonic growth (CN); **(C)** Crenulations of biotite flakes (PPL); **(D)** Coarse-grained porphyroclast of actinolitic hornblende wrapped around by mylonitic foliation (CN), **(E)** Radiading tremolite crystals in tremolite schist (CN). (**F**) Partially saussuritized plagioclase crystal with corroded outlines (CN); **(G)** Albite-orthoclase intergrowth in the form of flame perthite texture (CN); **(H)** Partially saussuritized plagioclase phenocryst (CN); **(I)** Palgioclase laths in the groundmass (CN). (Created by SmartSketch v. 4.0 software; https://smartsketch.software.informer.com/4.0/*).*

ii. *Metavolcano-sedimentary assemblage* (MVS).
  MVS is exposed in the central part on both sides of Wadi Um Laseifa and extends NW-SE for about 11 km from south to the northwest margin of the study area where it is intruded by MG and GD (Fig. 3B). These rocks are bounded from the east and west by MS and HS along thrust contact (Figs. 14 and 15G). This belt is dominant by MS in its eastern part and metavolcanic-volcanoclastic in its western sector. Different styles of structures such as regional foliation (Fig. 8B) minor normal faults and stretched pebbles (Fig. 15H) appear in these rocks. The MS comprise garnet-actinolite-biotite schist (Fig. 17B), actinolite-biotite schist (Fig. 17C) and actinolite schist with minerals of garnet, actinolite, and biotite that occurs in variable proportions in each variety in addition to quartz. Metavolcanics are represented by meta-andesites.

iii. *Hornblende schist (HS)*.
  HS is represented by belt in the extreme southwest thrusted northeastward over MVS along thrust faults characterized by mylonitic foliation (Fig. 14A, B) which under microscope show augen texture of quartz and amphiboles matrix elongated and wrapped around the actinolitic hornblende porphyroclasts (Fig. 17D). This belt is intruded from the western side by offshoots of younger granitoids (Fig. 15I). HS is characterized by dominant black colour that changed to bright yellow colour due to intensive shearing along internal thrusts which produce actinolite- tremolite schist (Fig. 17E). The absence of plagioclase as a major mineral and dominancy of quartz and hornblende may suggest a calc-silicate origin of HS which is supported also from remote sensing images (Figs. 7 and 8).

#### Granitoid rocks (MG, GD)

Intrusive rocks intrude HS and MVS from their western side by sharp contact dips eastwards (Figs. 3B and 6A). They have been differentiated to monzogranite (MG) and granodiorite (GD). Both types consist of plagioclase, quartz, orthoclase, biotite, with subordinate chlorite, epidote and sericite. The difference only is in increasing the percent of orthoclase in MG (Fig. 17F, G). The dykes have a main trend of NW-SE and rarely NE-SW. Acidic dykes are distributed as individual dykes (Fig. 15J) however; dyke swarm are recorded in the central part of the study area, which are well defined on the satellite images (Fig. 10A), where some extends for about 9 km in length and about 5 m in width (Fig. 10B, C,D). Acidic dykes consist of plagioclase and quartz phenocrysts embedded in a microcrystalline groundmass of the same minerals (Fig. 17H). The basic dykes (Fig. 15K) consist of plagioclase phenocrysts set in a microcrystalline groundmass of plagioclase, pyroxene and amphiboles (Fig. 17I). The Miocene sandstone consists of well sorted angular grains of quartz and exhibit cross bedding primary structures (Fig. 15L).

### Structural architecture

Based on all of our multiscale previous observations and findings, the structural framework of the studied area is established and included the following elements:

#### Thrust faults

Three major thrust faults are observed in Um Laseifa area and represent the tectonic boundaries between the metamorphosed rock units (e.g. Figures 14 and 15G). There were no noticeable field evidences for horizontal displacements on the plane of these faults. They constitute a thrust stack of NW-SE to NNW-SSE strike and SW dipping. The first one is recorded at the southeastern and northeastern sectors and mark the contact between the ophiolitic mélange, sheared serpentinites, metapyroxenite as hanging wall blocks and metavolcanics as a footwall block (Fig. 6A). The second fault is recognized in the central sector and characterized by a remarkable sharp lithological break between MVS and the MS (Fig. 15G). The third thrust fault lies at the western sector controlling the contact between the MVS as and HS (Fig. 14A). The thrust zones are characterized by a strong mylonitic foliation of well-developed papery appearance (Fig. 14B).

#### Strike slip faults

The rock outcrops are affected by five major strike-slip faults mainly of E-W trends. These faults are well recognized from remote sensing images (Figs. 6A, 8, 10, 11, and 12). Four of them are sinistral and the fifth is dextral. The first fault lies at the southern part, starts from the entrance of Wadi Um Gheig till the center of the study area with sinistral displacement, which resulted in disrupting all the rock units, folding axes and thrust planes for a distance of about 1.5 to 3 km and continues to southwest outside the study area (Figs. 6A and 12). The second one occurs at the southeastern part along Wadi Abu Lassaf with a NE-SW trend and 800 m dextral displacement, displacing the MS, MV and the inbetween thrust fault (Fig. 6A). The third one occurs to the north of Wadi Abu Lassaf and displacing the serpentinite mass for 1 km left lateral displacement (Fig. 6A). The continuation of the thrust contact occurs in the southern block of this fault to the northern block is not detected directly across the fault plane, but occurs at a distance of 4 km northwards due to a downward vertical displacement in the northern block associating the horizontal slip. The fourth fault lies at the western parts cutting across the whole rock succession up to the granitic rocks with 1 km sinistral displacement and splays in the granitic rocks to two faults (Figs. 6A, 8A, and 10A). The fifth fault has a sinistral displacement and occurs at the northern sector disrupting the MS, MV and the in between thrust fault (Fig. 6A).

#### Normal faults

Normal faults are restricted to the eastern and northeastern sectors of the study area. Three faults are detected along NW-SE to NNE-SSW trends and have downthrown sides towards the NE or SW (Fig. 9A). They are mostly related to red sea tectonics and control the areal distribution of the Miocene rocks, in addition to displacing the nonconformity surface along the Miocene/Basement contact at many locations (Fig. 9A).

#### Major folds

The study area is affected by two major folds identified in the ophiolitic mélange and the metavolcano-sedimentary rocks. The first fold is an asymmetric syncline occupying the southeastern and central parts till Wadi Wizr (Figs. 6B and 7A) and its axial plane has a NW-SE strike. The second fold is an asymmetric anticlinal fold affecting the MVS unit. Its axial plane strikes NW-SE parallel to the major thrusts (Fig. 13B).

**Fig. 18:**
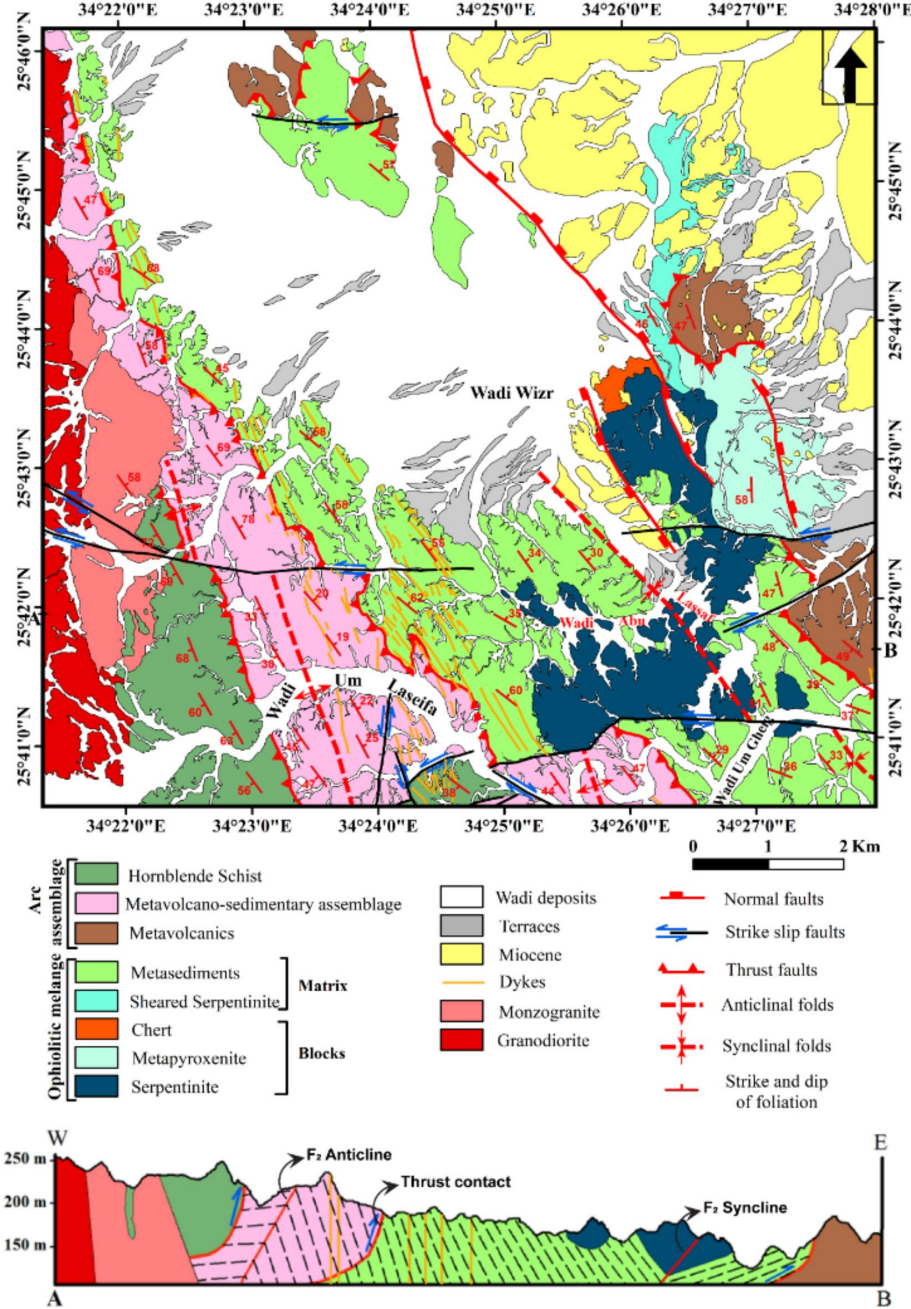
New geologic map of the Wadi Um Laseifa study area. The figure was created by ArcGIS Desktop 10.8. https://www.esri.com/en-us/arcgis/products/arcgis-desktop/overview, and SmartSketch v. 4.0 software; https://smartsketch.software.informer.com/4.0/.

Based on all of these integrated datasets and methods (Remote sensing, Field work, petrographic description) we built the final geological map of the study area (Fig. 18).

### Deformation phases and related mesoscopic structures

**Fig. 19:**
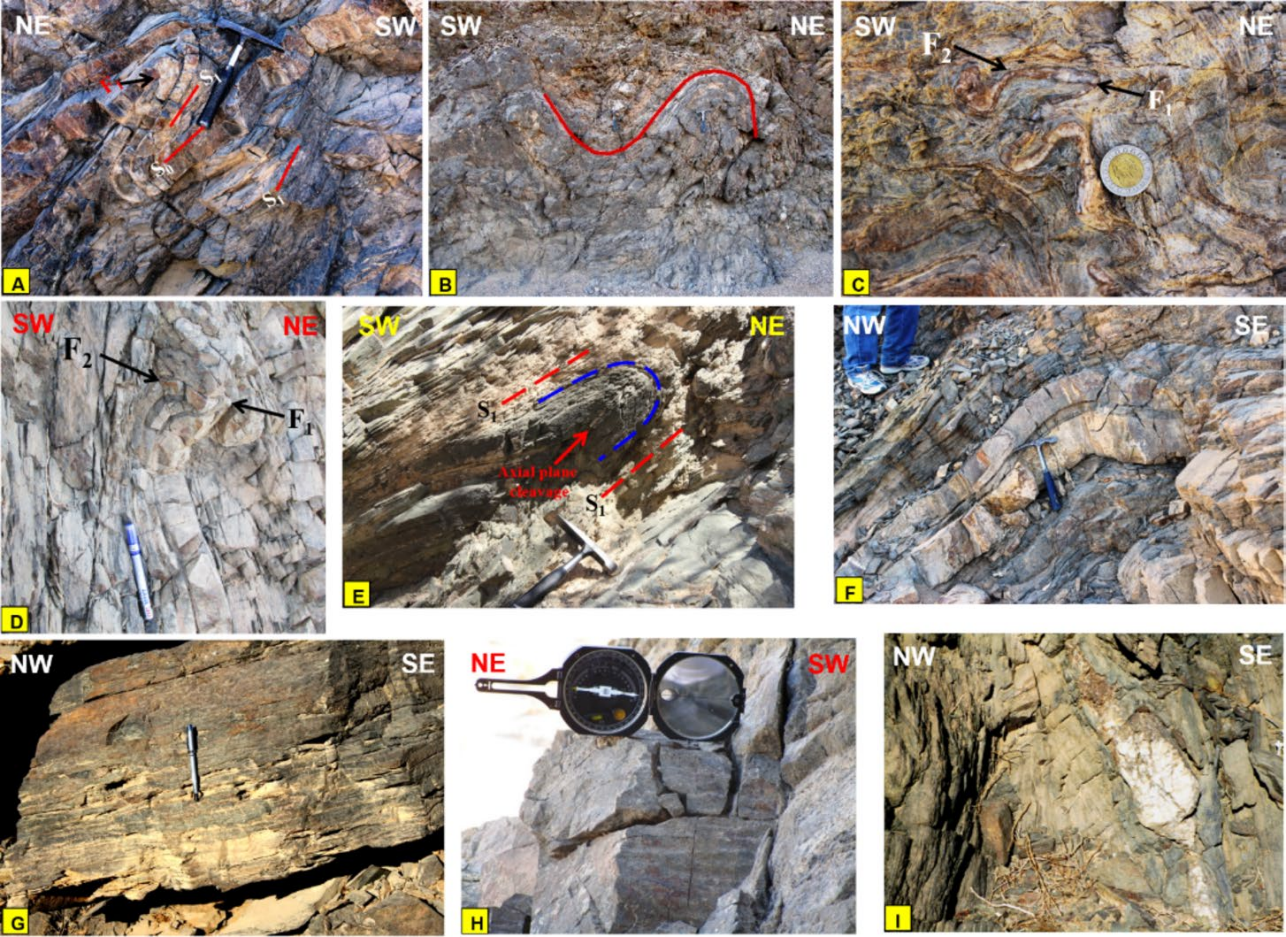
Field photographs showing (**A**) Tight overturned folds related to the first phase of folding. The figure also shows well developed S1 foliation parallel to S0 primary layering in the metavolcanoclastics, Wadi Um Laseifa. (**B**) Symmetrical syncline and asymmetrical anticline related to the second phase of folding well developed in metavolcanoclastics, Wadi Um Laseifa. (**C, D**) Interference pattern between F1 and F2 folding where a very tight F1 closure is refolded by moderately tight F2 fold. The interference pattern is developed in: (**C**) Metasediments in the center of the study area and (**D**) metavolcanoclastics in Wadi Um Laseifa. (**E**) Axial plane cleavage that is developed in the metasediments, Wadi Um Gheig. (**F**) Open (F3) anticline developed in the metavolcanoclastics, Wadi Um Laseifa. (**G**) Compositional lamination in the metasedimentary matrix, Wadi Um Gheig. (**H**) Crenulation lineation in the metavolcanoclastics, plunging SE at low angle. (**I**) Boudinage structure developed in the metasediments and plunging at low angle to NE, Wadi Um Gheig. (Created by SmartSketch v. 4.0 software; https://smartsketch.software.informer.com/4.0/).

**Fig. 20:**
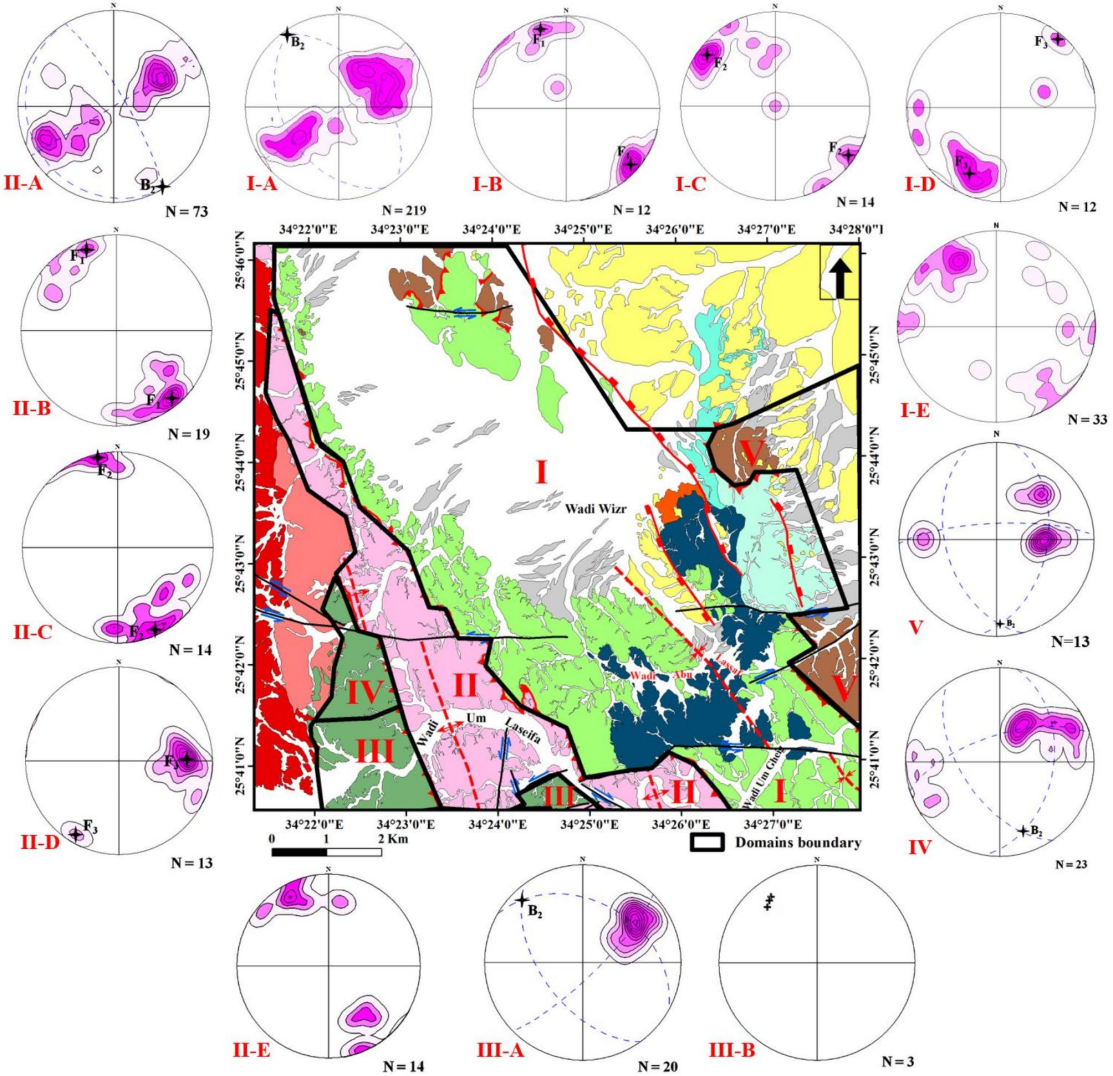
Map showing the distribution of the different structural domains in the study area with plotted schmidt equal area, lower hemisphere, stereographic projection of the structural elements in different domains. Contours are at 0,2,4,6,8,10,12% per unit area. The figure was created by ArcGIS Desktop 10.8. https://www.esri.com/en-us/arcgis/products/arcgis-desktop/overview, and SmartSketch v. 4.0 software; https://smartsketch.software.informer.com/4.0/.

Depending on field observations such as intensity of deformation, folding orientations and superposition relationships, the study area has been affected by three ductile deformation phases (D_1_, D_2_ and D_3_). Three folding phases (F_1_, F_2_ and F_3_) associated the different deformation phases have been observed on the mesoscopic scale (Fig. 19 A to F). They are observed mainly in the MS and MVS with a remarkable difference in their styles and attitudes of axial planes and folding axes. To support this classification, geometrical analysis has been performed on all measurements collected from the whole area for different structural elements. The study area has been divided into five domains **(I**,** II**,** III**,** IV**,** V)** following criteria presented in^108,115,116^, taking in consideration the presence of structural contacts between the different rock units (Fig. 20). Domian I represents the major synclinal fold affecting the ophiolitic mélange association around Wadi Wizr, Domain II covers the anticlinal fold in the MVS. Domains III and IV cover the northern and southern sectors of HS belt. Domain V covers the MV. Domains III to V are remarkably less foliated, in addition to in domains III and IV, most exposures are highly weathered, disrupted by numerous internal thrusts, and intensively intruded by granitic offshoots. Therefore, there was a considerable difficulty to collect structural data from these domains and also, they don’t reflect the effect of folding on the major scale. However, the available data have been collected and presented. The orientations all structural elements as determined from the geometrical analysis of the field measurements are summarized in Table 2.

**Table 2:** Summary of the geometrical analysis results.

Domain	Regional foliation		Major folding axis	Axes of minor folds					
				F_1_		F_2_		F_3_	
D I	N41˚W/48˚SW	N38˚W/44˚NE	4˚ / N35˚W	12˚/ N17˚W	10˚/ S49˚E	11˚/ N52˚W	7˚/ S56˚E	7˚/ N40˚E	22˚/ S26˚W
D II	N30˚W/48˚SW	N26˚W/67˚NE	0˚/N31˚W	11˚/ N20˚W	10˚/ S39˚E	4˚/ N13˚W	6˚/ S26˚E	28˚/ N88˚E	11˚/ S30˚W
D III	N40˚W/56˚SW								
D IV	N38˚W/57˚SW								
D V	N45˚W/54˚SW								

#### D1 phase

The D_1_ has resulted in the development of F_1_ folds which are of very tight isoclinal intrafolial, tight recumbent and overturned styles (Fig. 19A), and plunging mainly to the NW and SE at low to moderate angles. F_1_ folds show axial planes lying parallel or at low angle to the surrounding foliation and they have a small interlimb angle (not exceeding 30˚). In domain I, the orientation diagram of the axes of the first phase folds (F_1_) defines two maximum concentrations with a mean fold axis attitude (12/N17˚W and 10/S49˚E) (Fig. 20I-B; Table 2). In Domain II, the axes of F_1_ phase have two main trends (Fig. 20 II-B, Table 2). F_1_ folds are not traced in Domains III, IV, and V but the angle between the two foliation poles concentration in domains IV and V is small (37 and 28 respectively) which points to the presence of some tight overturned F_1_ folds within the data (Fig. 20 IV and V).

Foliations (S) in general comprises slaty cleavage, schistosity, strain-slip cleavage, axial plane cleavage, and mylonitic foliation. S_1_ is represented by the regional schistosity and axial plane cleavage of F_1_ folds, mainly of NW-SE strikes and dip to the NE and SW at moderate angles (Fig. 20I-A, II-A, III-A, IV, V, Table 2). Schistosity foliations are developed parallel or at low angle to the (S_0_) primary bedding, laminations that are well recognized in MS (Fig. 19G), and axial plane cleavage developed parallel to the axial planes of F_1_ tight intrafolial folds (Fig. 19E). Linear elements in domain I show distribution of data around three concentrations; plunging NW, SE and NE at moderate angles, almost coaxial with the F_1_, F_2_, F_3_ orientations (Fig. 20I-E), whereas in domain II they are coaxial to F_1_ and F_2_, showing two maximum concentrations of NW and SE trends and gentle to moderate plunges (Fig. 20 II-E).

#### D2 phase

D_2_ deformation is the major event responsible for major folding, thrusting and associated structures (Fig. 18). Also, F_2_ folds are developed on the outcrop scales, attaining upright folds of moderately tight symmetric and asymmetric styles (Fig. 19B), chevron folds and a mesoscopic scale fold that can be traced easily on the Google Earth satellite images (Fig. 13A, B). They are plunging at low to moderate angles (10˚ to 50) to the NW and SE almost coaxial to F_1_ folds. These folds are of parallel geometry showing a uniform true thickness of individual layers at the limbs and crests of these folds. The parallelism of the axial planes and folding axes and similarity in folding style between the major folding and (F_2_) minor and mesoscopic folds indicates that major folds are developed through D_2_ phase. The superposition relationship between F_2_ and F_1_ phases is represented by interference patterns, developed in the metasediments (Fig. 19C) and metavolcanoclastics (Fig. 19D). In these interference patterns, (F_1_) very tight fold closures and (F_1_) axial planes are clearly refolded by the moderately tight (F_2_) folds, attaining hook shape geometry of type 3 interference pattern of^116^.

In domain I, the plotting of the poles to the main foliation (S_1_) exhibits rotation of data around a great circle with two maximum concentrations (Fig. 20I-A; Table 2). The pole to this great circle indicates the axis of the major fold (B_2_) plunging 4˚ on a bearing N35˚W. The mesoscopic folds (F_2_) also exhibit two maximum concentrations with a mean fold axis attitude (11˚/ N52˚W and 7˚/S56˚E) which are almost coaxial with F_1_ folds (Fig. 20I-C). In domain II, the plotting of the poles to S_1_ foliation data (Fig. 20II-A, Table 2) defines the statistical orientation of the axis of the major fold B_2_ (0˚/N31˚W). F_2_ mesoscopic folds has axes attitudes (4˚/ N13˚W and 6˚/S26˚E) (Fig. 20II-C). The poles to S_1_ foliation in domain III show a tendency of rotation of data around a great circle with one elongated maximum concentration (Smean: N40˚W/56˚SW) (Fig. 20III-A). The attitude of the pole to that great circle is 12˚/ N44˚W, which is conformable with F_2_ fold axes in the previous domains. From plotting the poles to S_1_ foliation in Domain IV, we attain a pole attitude 20˚/S18˚E (Fig. 20IV), which is almost parallel to the axes of the F_2_ outcrop folds in domains I and II. The foliation data in this domain is shown (Table 2) with mean attitude N38˚W/57˚SW. The fact that the metavolcanics (Domain V) are related to the same tectonic setting of the other metamorphosed rock units in the study area, and the parallelism of its foliation (N45˚W/54˚SW) to those in the eastern limb of the major syncline, indicates that they must be affected by the same successive deformations affected the other rock units. The orientation diagram of the poles to the foliation (S_1_) in the metavolcanics shows a rotation of data along a great circle pattern with three concentrations (N45˚W/54˚SW, N-S/41˚W, N0˚E/64˚E) (Fig. 20V; Table 2). The pole of the great circle defines the statistical orientation of an axis of folding 13˚/ S2˚E (Fig. 20V), running parallel to F_2_ folds in domains I and II.

The S_2_ fabric is generally of NW-SE strike and dip to the SW at moderate to high angles, coeval with the growth of F_2_ folds and overprints S_1_ regional schistosity. The parallelism of mylonitic foliation and thrust planes to the axial planes of the F_2_ major and minor folds pointing to the fact that both of them are coeval and the mylonitic foliation (Fig. 14B) is an S_2_ fabric element. Linear elements represented by stretched pebbles (Fig. 15H) and crenulation lineations (Fig. 19H). They are plunging toward NW at moderate angles, coaxial to F_2_ folds (Fig. 20 III-B).

#### D3 phase

The D_3_ phase produced open symmetric and asymmetric F_3_ folds of vertical to sub-vertical axial planes striking NE-SW (Fig. 19F). The fold axes plunge mainly toward SW and occasionally toward NE or ENE with angles range from 15˚ to 50˚. The rotation of F_3_ data is due to that they are formed on the limbs of older folds. These folds are parallel in style; having uniform true thickness of individual layers in both their limbs and crests. In domain I, the orientation diagram of the F_3_ folds axes defines two maximum concentrations with attitudes 7˚/N40˚E and 22˚/S26˚W (Fig. 20I-D). In domain II, the third phase is characterized by two concentrations (28˚/N88˚E and 11˚/S30˚W) (Fig. 20II-D). F_3_ folds are associated with NE-SW subvertical S_3_ axial plane cleavage and L_3_ boudinage (Fig. 19I) and pencil structures.

## Discussion

The Neoproterozoic ophiolites in the Central Eastern Desert (CED) have been extensively studied over the past few decades, primarily focusing on their origin and tectonic setting through mineralogical, petrological, and geochemical methods^29,73–81^. These valuable contributions helped in advancing our understanding of the region’s geology. Building upon these findings, our study introduces additional insights using advanced remote sensing datasets, such as Prisma hyperspectral and PlanetScope data (3 m), complemented by extensive fieldwork, structural mapping, and petrographic analysis. To mitigate the limitations or potential sources of error (e.g., geometric distortions) associated with remote sensing analysis, we used several datasets with different spatial and spectral characteristics to ensure data reliability. Additionally, we conducted field validation of remote sensing-derived features to assess the accuracy of geological interpretations. This approach allowed us to address unresolved mapping challenges, producing a refined geological map that identifies new lithologic units and rock contacts with enhanced accuracy.

Previous research addressed the deformation history of CED ophiolites, with studies indicating that NE-SW shortening during the arc-back-arc stage resulted in a single ductile deformation phase (D1)^84,93,117^. Our analysis, however, reveals a more complex deformation history. Through comprehensive geometrical analysis of numerous structural measurements across different rock units and superposition relationships between various folding generations, we identified two successive, coaxial ductile phases (D1, D2) within this initial NE-SW event. Additionally, we identified a subsequent ductile phase (D3) resulting from NW-SE shortening. These findings were further validated by tracing and resolving distinct structural elements, including foliations and lineations, associated with each deformation phase.

### Geological mapping

The present study introduced several enhancements compared to previous efforts in terms of the classification of the major rock units, their distribution and type of contacts in between and structural framework. According to our findings, the stratigraphic succession is classified into: ophiolitic mélange (serpentinites, metapyroxenite, and chert blocks dispersed in matrix of sheared serpentinites and metasediments), metavolcanics, metavolcano-sedimentary rocks and hornblende schist (Fig. 18). This succession was intruded by granodiorite, monzogranite, dykes and covered by Miocene sediments (Fig. 18). These rock units are superbly differentiated on the Sentinel 2 images, carefully checked during fieldwork and confirmed by extensive petrographic investigations. According to our knowledge, the hornblende schist, metapyroxenite belt and chert block are mapped in this study for the first time (Fig. 18). The metasediments, metavolcano-sedimentary, and hornblende schist belts differentiated in this study were group as metavolcano-sedimentary schist by El-Hebiry^56^. The metasediments unit extension mapped here and surrounding the serpentinites were different from Fowler et al.^57^ who mapped it as metavolcanics. The thrust contacts separating the different rock units and well defined in the field by their papery mylonitic foliation (Fig. 14A, and B) can be relatively compared to the contacts mapped by Asran^54^ and El-Hebiry^56^ who mapped thrust faults bounding the main serpentinite mass from its southwestern side. On the other hand, these contacts cannot be compared with the contacts mapped by El Bahariya et al.^58^. who mapped them as normal faults and Abdeen et al.^90^ who mapped it as oblique slip faults. This contact was indicated as thrust contact related to the obduction of ophiolitic in other places in the CED^93,100,118,119^. The contact between the granites in the western part and the adjacent rocks was mapped by Asran^54^ and Farahat et al.^92^ as a fault contact, whereas this contact is intrusive as indicated by the presence of granitic offshoots in the country rocks (Fig. 15I).

### Deformation history

**Fig. 21:**
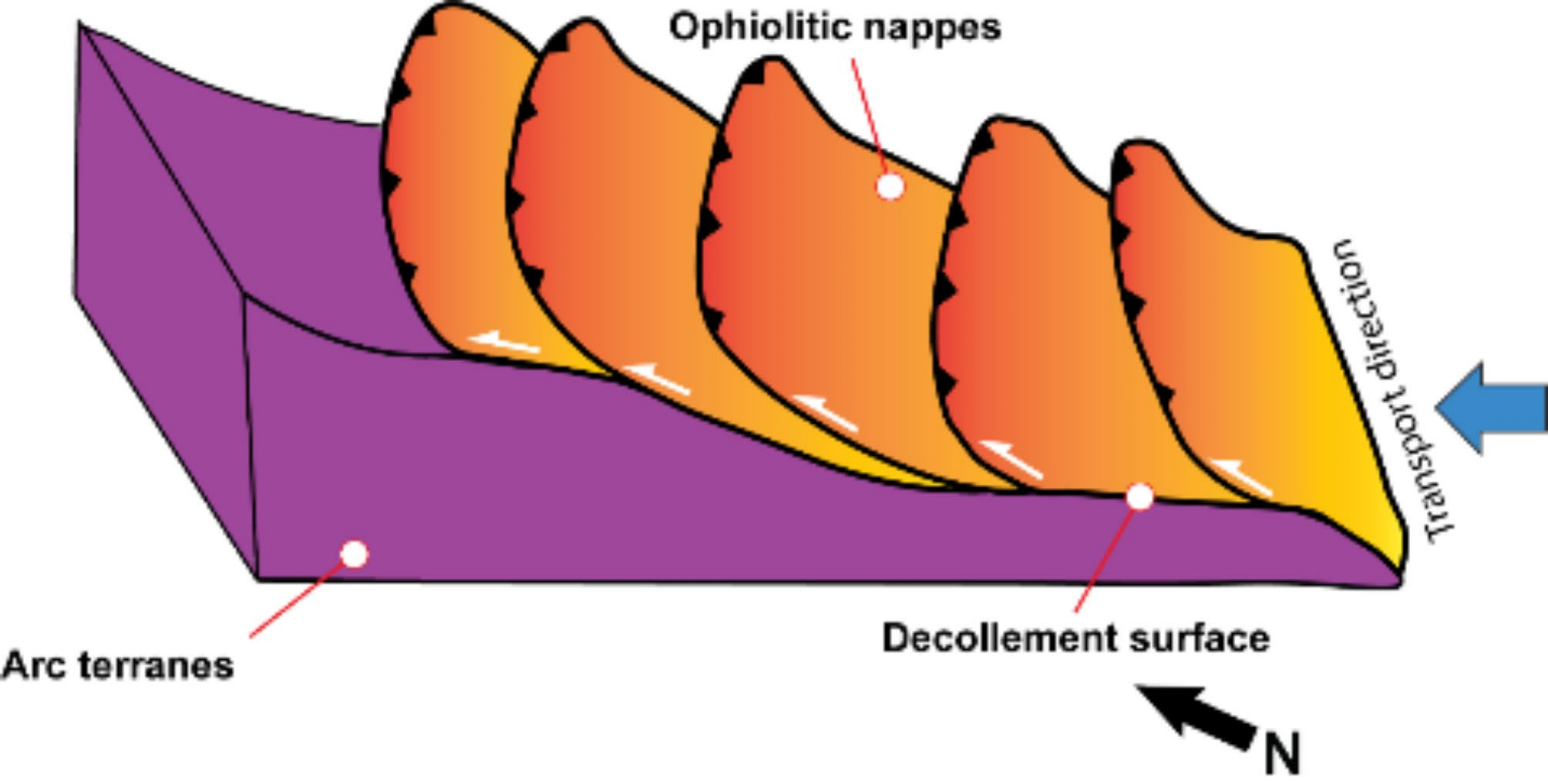
3D sketch diagram showing a NW-ward thrusting of ophiolitic nappes over arc terranes.

**Fig. 22:**
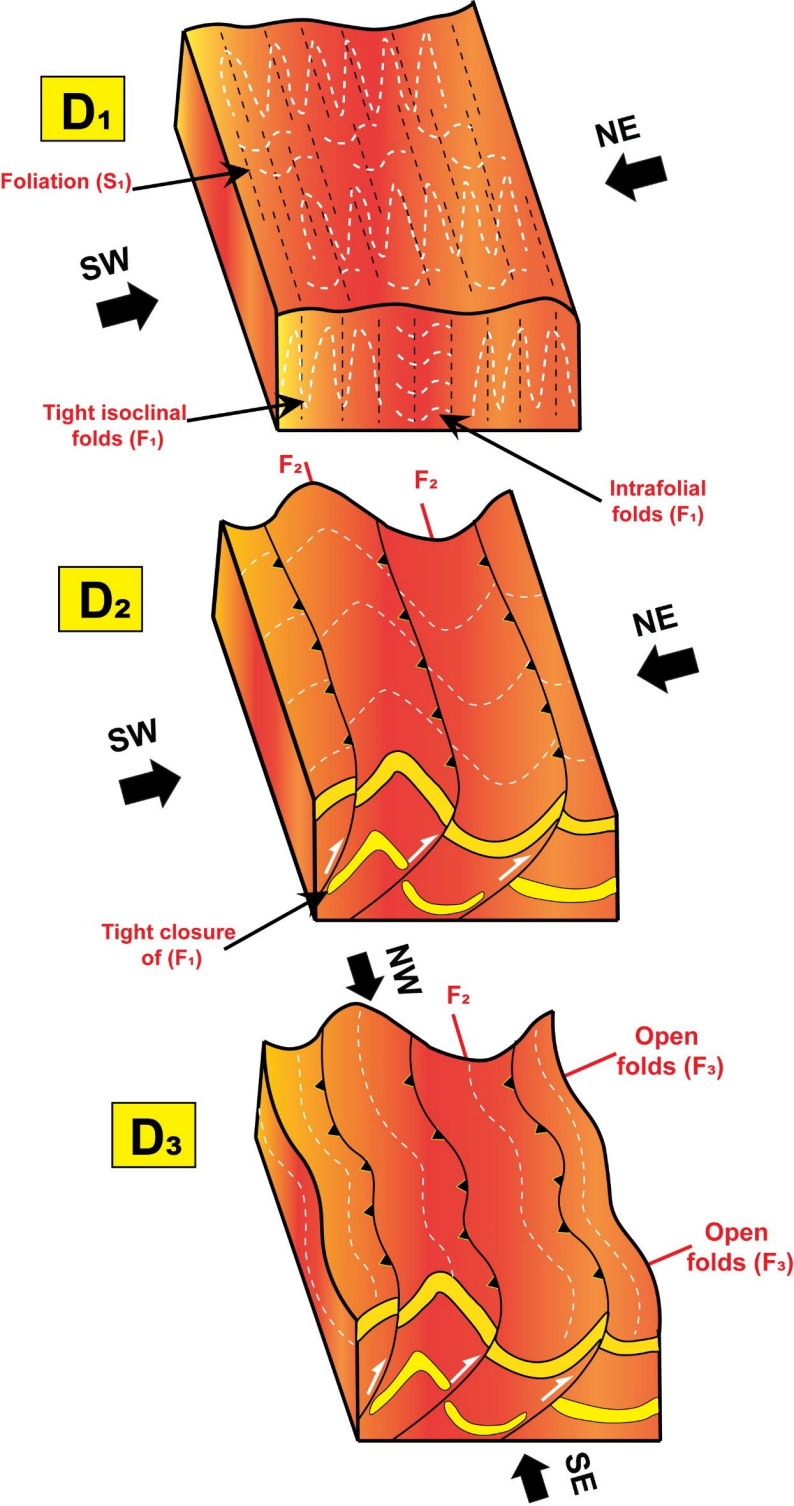
A tectonic evolution model illustrating the successive deformation phases (D1, D2, D3) occurred in the arc accretion stage (680 ± 10 Ma, Bregar et al.,^126^) and affected on the Neoproterozoic rocks (in the study area).

The low grade ophiolitic rocks in the ED were considered as remnants of oceanic crust formed during the opening of the Mozambique Ocean and Rodinia break between (850 and 700 Ma)^74.80,120,121,122^. The tectonic evolution of the ANS starts with the breakup of this supercontinent^123^. The arc accretion stage starts with the closure of the Mozambique Ocean (750–650 Ma^124,125^) and subsequent formation of the super continent Gondwana due the collision between East and west Gondwana (635 Ma). This stage results in the thickening of the ANS, oblique convergence and formation of island arcs. Also, this results in the formation of thin-skinned tectonic style in ophiolitic and volcano-sedimentary sequences with low/mid metamorphic conditions of 500º C and 0.5 GPa^61^ (i.e. low angle thrusting in the ophiloitic assemblage and thrusting over the arc terranes in NW direction (Fig. 21). Ophiolites together with arc assemblage and calc alkaline granitoids were formed during arc/back arc stage occurred between 750 and 620 Ma in the CED^40^, while the accretion of arc relics and remnant oceanic crust took place in EL-Sibai area to the west of the study area at around 680 ± 10 Ma^126^. Based on previous geochemical studies, the ophiolitic rocks here were considered to represent arc-back-arc assemblage^82,92,127^. The hornblende schist mapped here most probably represents metamorphosed pelagic carbonate sediments of a back-arc basin. The tectonic evolution of the study area can thus be explained as arc-back-arc deformation and crustal shortening events, marked by a NE-SW compressional stresses that produced major NW-SE thrust stacking, regional foliation and major folding during the second phase of deformation (D_2_) (Fig. 22). This conclusion agrees with Fowlar et al.^57^ who related the formation of NW-SE upright folds (F_2_) to that event. Abd El-Wahed^118^ related the SW-dipping thrusts in volcaniclastic metasediments along Wadi Sitra to the southwest of the study area to ENE–WSW compression event during the obduction of ophiolites and the NW-trending macro and mesoscopic folds are concurrent with the NE-ward thrusting episode which all followed the NNW directed stacking event (Fig. 21). Shortening stresses in ESE- to ENE are evidenced throughout the CED and resulting also in the formation of NNW- to N-trending folds and superimposed on early NNW-directed thrusting episode^90,128–130^ (Fig. 21).

Due to intensive deformation affecting the study area (Fig. 21). Deformation phases have addressed in different scenarios. The NW regional folding, foliation and isoclinals intrafolial minor folds was considered as D_1_ phase and thrust faults and associated isoclinals folds were related to a D_2_^58^. Our field evidences showed that mylonitic foliation and thrust planes are parallel to the axial planes of the F_2_ major and minor folds which support that both of them are coeval. So, D_1_ regional folding and D_2_ thrusting discussed in^58^ are equivalent to the D_2_ of the present study (Fig. 22). The major folds and thrust were considered as the first phase of deformation in the study area^56^. This phase of deformation can be compared with D_2_ in the present work. A brittle D_3_ phase responsible for normal and strike slip faulting was proposed by El-Hebiry^56^ and El-Bahariya et al.^58^, meanwhile D_3_ is not assigned as a separate phase in the present study. In conclusion, our new data confirmed the presence of an earlier D_1_ phase superimposed by the major D_2_ phase that was noticed in most previous works (Fig. 22). The D_1_ earlier phase of folding was recorded by Fowlar et al.^57^ but with completely different trends (N to NE and S to SW) comparing to our findings (NW, SE trends and gently plunging in both directions) (Fig. 20). As far as we know, the NE-SW trending folds that produced by D_3_ phase in this work (Fig. 22) were rarely delineated except of^89.90^. The series of strike slip faults with almost E-W trends that cut through the whole succession was considered D_4_ brittle deformation phase which associated with low crustal levels exhumation and cooling followed the emplacement of post-tectonic granitic intrusions^93^.

## Conclusions

This study represents the first comprehensive investigation of the Wadi Um Laseifa area in the Central Eastern Desert of Egypt, utilizing a multidisciplinary approach that integrates remote sensing data (Sentinel-2, PlanetScope, and PRISMA) with detailed field observations and laboratory analyses. The study area, renowned for hosting significant occurrences of ophiolitic rocks within the ancient Arabian Nubian Shield, provides a critical window into the region’s complex tectonic history. The integrated approach has resulted in an updated geological map with improved delineation of lithological contacts and structural features. These findings contribute significantly to our understanding of the regional tectonic framework of the ANS, an area that continues to demand extensive research. The current research concluded the following:

1. The exposed rock units in the Wadi Um Laseifa area are petrographically differentiated into ophiolitic mélange association of ophiolitic blocks (serpentinite, metapyroxenite, metagabbro and chert) dispersed in a mélange matrix (metamudstone, metasiltstone, metagreywacke, and schist), arc assemblage (metavolcanics, metavolcano-sedimentary sequence and hornblende schist), intrusive granitoids, dykes and Miocene sediments. The metamorphosed rock units are affected by a thrust stack of three major faults controlling their contacts; striking NW-SE to NNW-SSE and dip steeply to the SW.
2. Two major folds are present in the study area; a major anticline affects the metavolcano-sedimentary rocks and a major syncline affects the ophiolitic mélange. Both folds have axial planes striking NW-SE and fold axes gently plunging NW. The study area is traversed by a series of E-W or ENE-WSW strike slip faults mainly of left lateral movements and also affected by three major NW-SE normal faults with down thrown sides towards NE or SW which are related to the Red Sea tectonics controlling the distribution of the Miocene deposits in the study area.
3. The tectonic evolution of the study area can be regarded as arc-back-arc deformation and crustal shortening events. The metamorphosed rocks were affected by three ductile deformation phases (D_1_, D_2_, D_3_), resulted in major folding, thrusting and different generations of minor folds, foliation and lineation. D_1_ is represented by minor NW-SE isoclinals intrafolial folds. D_2_ is a major event produced NW-SE trending major folds and thrust faults. D_1_ and D_2_ phases are nearly coaxial. D_3_ produced NE-SW trending open minor folds. Each phase is associated by its characteristic foliation and lineation.
4. Our methodology effectively integrated remote sensing, fieldwork, and petrographic studies to enhance lithological and structural mapping. Remote sensing provided an initial overview, enabling the identification of rock units and guiding fieldwork. Ground-truthing refined these interpretations, while petrographic analysis provided critical micro-scale validation. This multi-faceted approach, involving cross-validation at multiple scales, significantly reduced ambiguity and ensured a more comprehensive and reliable understanding of the study area. This study’s methodology offers a valuable framework for geological mapping in other arid, structurally complex regions, providing insights that can enhance and refine regional tectonic models. Future applications of this approach will support more accurate geological interpretations.

## Data Availability

The datasets used and/or analyzed during the current study are available from the corresponding author upon reasonable request.
